# Specificity of a protein–protein interface: Local dynamics direct substrate recognition of effector caspases

**DOI:** 10.1002/prot.24417

**Published:** 2014-10-19

**Authors:** Julian E Fuchs, Susanne von Grafenstein, Roland G Huber, Hannes G Wallnoefer, Klaus R Liedl

**Affiliations:** Institute of General, Inorganic and Theoretical Chemistry, Center for Molecular Biosciences Innsbruck (CMBI), University of InnsbruckInnrain 80/82, A-6020, Innsbruck, Austria

**Keywords:** protein–protein interface, caspase, substrate recognition, specificity, conformational selection, local dynamics

## Abstract

Proteases are prototypes of multispecific protein–protein interfaces. Proteases recognize and cleave protein and peptide substrates at a well-defined position in a substrate binding groove and a plethora of experimental techniques provide insights into their substrate recognition. We investigate the caspase family of cysteine proteases playing a key role in programmed cell death and inflammation, turning caspases into interesting drug targets. Specific ligand binding to one particular caspase is difficult to achieve, as substrate specificities of caspase isoforms are highly similar. In an effort to rationalize substrate specificity of two closely related caspases, we investigate the substrate promiscuity of the effector Caspases 3 and 7 by data mining (cleavage entropy) and by molecular dynamics simulations. We find a strong correlation between binding site rigidity and substrate readout for individual caspase subpockets explaining more stringent substrate readout of Caspase 7 via its narrower conformational space. Caspase 3 subpockets S3 and S4 show elevated local flexibility explaining the more unspecific substrate readout of that isoform in comparison to Caspase 7. We show by *in silico* exchange mutations in the S3 pocket of the proteases that a proline residue in Caspase 7 contributes to the narrowed conformational space of the binding site. These findings explain the substrate specificities of caspases via a mechanism of conformational selection and highlight the crucial importance of binding site local dynamics in substrate recognition of proteases. Proteins 2014; 82:546–555.

## INTRODUCTION

Protein–protein interactions are of crucial importance for many biological processes.^1^ Still, (multi)specificity of the large interaction sites in protein–protein interfaces is poorly understood.^2^,^3^ A static view of protein–protein recognition is more and more found insufficient to describe the dynamic conformational states of both binding partners.^4^–^7^

A binding paradigm of conformational selection has been found helpful in interpretation of complex protein–protein recognition patterns.^8^ Thereby, one binding partner selects the most appropriate conformation of the other partner out of a pre-existing ensemble of multiple conformations.^9^ The conformational equilibrium is shifted toward the bound state upon the binding event.

Proteases are a especially well-suited protein family to study protein–protein interactions.^10^ Proteases recognize and cleave protein and peptide substrates at a well-defined position in a substrate binding groove.^11^ A plethora of experimental techniques allows to gain experimental insights into protease-substrate recognition.^12^–^14^ Recently, a quantitative specificity index for proteases and their subpockets “cleavage entropy” has been established.^15^ Therefore, protease specificity can be experimentally traced and quantified as well as mapped back to particular regions of the binding site, providing unique insights into protein–protein recognition.

Caspases (cysteine-dependent aspartate-specific proteases) are a group of intracellular proteolytic enzymes relying on a catalytic cysteine—histidine dyad.^16^ Their biological role comprises the propagation of programmed cell death (apoptosis), proliferation and inflammation.^17^ Because of their central role in apoptosis, caspases have been extensively studied with regard to their potential as drug targets, e.g., in chemotherapy.^18^,^19^ Members of the caspase family are divided into caspases activating the cell death machinery (initiator Caspases 2, 8, 9, and 10) and executioners of programmed cell death (effectors: 3, 6, and 7).^17^

Especially Caspases 3 and 7 share pronounced sequence similarity amongst effector caspases, whereas the homologous Caspase 6 diverged more over evolution,^20^ which is also reflected in its different substrate readout.^21^ Caspases 3 and 7 share highest sequence similarity on the full length protein amongst all pairs of apoptosis signaling caspases (see Yoshimori *et al*.^20^ for a sequence alignment). On a structural level, the catalytic cleft of caspases is surrounded by two flexible flaps that are longer in Caspases 3 and 7 compared to other members.^21^ These surface loops were described to play a central role in the activation of caspases from the zymogen procaspase^22^ via proteolytic cleavage. Different mechanisms of these activation steps and its impact on the active site geometry are discussed in literature.^23^–^25^ Even small molecules are described as activators of effector caspases leading to apoptosis in cancer therapy.^26^ The dimer interface of Caspase 3 forms a bifunctional allosteric site allowing both activation and inactivation.^27^ After proteolytic activation caspases comprise a large and a small subunit forming a β-sheet sandwiched between α-helices. The central β-sheet is elongated with another subunit to form a stable homodimeric assembly.

As structures of Caspases 3 and 7 are overall highly similar^28^ and both enzymes were shown to have virtually indistinguishable specificity for tetrapeptides^25^ as well as comparable half-lives,^29^ they were long thought to be redundant enzymes. Both Caspases 3 and 7 cleave at the canonical recognition site DEVD in the P4–P1 region derived from the substrate Poly(ADP-ribose) polymerase PARP,^21^ whereas caspases exhibit less stringent substrate readout in the P′-region. No difference between substrate specificities of Caspases 3 and 7 was found via positional scanning peptide libraries,^30^,^31^ only subtle differences were described in HPLC turnover measurements of flourescence-labeled substrates.^32^ As Caspases 3 and 7 possess highly similar substrate specificities, they were thought to exhibit overlapping, if not redundant roles in cells.^33^ As a Caspase 3/7 double knock-out mouse died immediately after birth, a surrogate function was assumed.^34^ Surprisingly, distinct phenotypes were observed on knockout mice of Caspases 3 or 7.^35^,^36^

In recent years, novel technologies in protease substrate profiling revealed differences in the substrate spectra of Caspases 3 and 7^37^: The database CASBAH was established to gather information on known caspase substrates.^38^ In 2008, Walsh *et al*. could show, that Caspases 3 and 7 indeed cleave nonoverlapping substrates, hence are nonredundant, functionally distinct proteases.^39^ Furthermore, they found Caspase 3 to be generally more promiscuous than Caspase 7, in analogy to PICS assays revealing more substrates for Caspase 3 than for Caspase 7.^12^ Still, caspases belong to the proteases showing highest substrate specificity (Fuchs et al., submitted for publication).^17^ The overlap and differences in substrate spectra of Caspases 3 and 7 were further analyzed by Demon *et al*. revealing complex statistical recognition patterns beyond the canonical tetrapeptide.^40^ Rules rationalizing substrate readout of caspases on the secondary, tertiary and quaternary structural level have been established,^41^,^42^ as well as a distinct network of protein interactions between caspase substrates.^43^ Such rules of different levels of complexity have been employed in several techniques to computationally predict cleavage sites of caspases.^44^–^46^

Besides knowledge-based prediction of cleavage sites, computational approaches in the field of caspases mainly comprise docking efforts to improve affinity^20^ and selectivity^47^ of known peptide ligands as well as their structure-based design.^48^ Modeling approaches were also applied to rationalize substrate specificities inside and outside the tetrapeptide recognition motif of caspases.^40^,^49^,^50^

Although Fang *et al*. revealed the crucial importance of flexible adaption of the protease to a peptide ligand,^50^ aforementioned computational approaches are based on a simplistic rigid view of the protein. Molecular dynamics (MD) simulations are capable of incorporating a large part of the accessible conformational ensemble of proteins into computational predictions. These dynamic factors are known to be crucial for protein function and its prediction.^51^ Hence, theoretical studies covering protein flexibility are computationally demanding, but highly desirable for the functional understanding of caspase recognition. Published MD studies focus on the dynamics of procaspase 3 activation^52^,^53^ as well as the differences between monomeric and dimeric assembly of caspases.^54^ A recent study investigates allosteric modulation of Caspase 3 by a combined approach covering mutational as well as MD studies, proves subtle structural differences to be crucial for catalytic activity of caspases.^55^ MD studies have also been successfully employed to model fluorescence lifetime imaging assays of Caspase 3 *in silico*.^56^

In our study, we performed data mining and MD studies to elucidate the origin of subtle differences observed in the substrate specificities of Caspases 3 and 7. We found that conformational dynamics are a major driving force in the substrate recognition of these effector caspases.

## MATERIALS AND METHODS

### Data mining of protease substrate databases: Cleavage entropy

To quantify substrate specificity of caspases, we extracted experimental cleavage information from the MEROPS database,^57^ as this general protease database was found to contain the most comprehensive set of annotated cleavage sites for caspases. All apoptosis signaling caspases with more than 50 annotated substrate sequences were included in the analysis (database accession 22.05.2012, MEROPS 9.6). Substrates were aligned in respect to the scissile bond and cleavage entropy scores were calculated for the adjacent protease subpockets as described in Fuchs *et al*.^15^ These cleavage entropies depict a measure of specificity for individual protease subpockets in the range of 0 (specific) to 1 (unspecific) and hence allow to correlate specificity intuitively to any other descriptor.

### MD simulations

We selected the inhibitor-bound structures of Caspases 3 and 7 showing the highest resolution from the PDB (PDB: 1PAU, 1F1J) as starting structures for our simulations^28^,^58^ with comparable resolutions of 2.5 and 2.35 Å, respectively. As the structure of Caspase 3 contained a monomer of the protease in the asymmetric unit, we constructed the biological dimeric assembly via simple symmetry operation around the twofold symmetry axis using Pymol.^59^ The dimeric state was already given for the Caspase 7 crystal structure and used as is. Simulation of the biologically relevant dimeric state removes artifacts introduced by the exposure of the dimer interface to the solvent leading to artificially increased flexibility and disintegration of the active site as observed by Sulpizi *et al*.^54^

Both starting structures contained an acetyl-DEVD-aldehyde adduct covalently bound to the active site cysteine in the P4–P1 region. This bond was broken artificially and the free inhibitory peptide capped with a C-terminal aminomethyl group to avoid perturbations by a free negatively charged C-terminal. Afterward, a local energy minimization of the aminomethyl group was carried out in MOE.^60^ The N-terminal of the ligand was used as is, capped with an acetyl group. The cocrystallized sulfate ion per subunit in the Caspase 7 structure was removed to ensure comparability between Caspases 3 and 7 simulations.

Protonation was carried out applying MOE's Protonate3D function^61^ and adjusted manually to ensure physiological protonation states. In addition to the complexed structures, we also generated holo systems by artificially removing the peptide ligand from the active site. Hence, we resulted in four topologies: Caspase 3 in ligand-free dimeric holo state CASP3_holo_ and ligand-bound dimeric complex state CASP3_com_ and the respective two systems for Caspase 7 CASP7_holo_ and CASP7_com_.

In addition to all water molecules resolved in the X-ray structures, resulting systems were solvated using tleap of the AmberTools package^62^ with a minimum wall distance of 12.0 Å with TIP3P water molecules.^63^ Simulations were carried using the AMBER10 package^62^ using the ff99SBildn parameter set for proteases and substrates including the capping groups.^64^ A uniform neutralizing plasma for Particle Mesh Ewald simulations was applied to neutralize the total charge of the periodic simulation box.^65^

After minimization with harmonic restraints on protein heavy atoms, the systems were gradually heated from 100 to 300 K over 200 ps in NVT ensemble (see Wallnoefer *et al*.^66^ for more details). A density equilibration over 1 ns was performed, followed by free simulations of the systems in NpT ensemble over 50 ns to ensure reasonable equilibration of the simulated systems. Production runs were carried out at 300 K using the Langevin thermostat^67^ at 1.0 bar with 8.0 Å nonbonded cutoff. A 2.0 fs time step was possible due to usage of the SHAKE algorithm.^68^ Snapshots were saved to trajectory every 500 steps or equivalent 1 ps for analysis.

Trajectories were analyzed using ptraj (version 4/2010) from AmberTools.^62^ Positional fluctuations of C_α_-atoms were calculated to assess stability and flexibility of protein structures over simulation times. Residue-wise B-factors were calculated as a measure of local protein flexibility. Distances and interactions of protein and ligand were likewise calculated by ptraj. For hydrogen bonding quantification default definitions were applied, the maximum heavy atom distance was hence set to 3.0 Å, the maximum angle between donor, hydrogen and acceptor to 135°.

### *In silico* mutations

As analysis of protein flexibility revealed Pro-235 as crucial factor in binding site rigidity of Caspase 7, an exchange mutation of Pro-235 in Caspase 7 to Ser-343 of Caspase 3 was carried out *in silico*. After superposition of Caspases 3 and 7 in Pymol^59^ (root mean square deviation of C_α_-atoms = 0.57 Å), the two residues in the S3 subpocket were exchanged to yield starting coordinates for the simulations CASP3-Pro_holo_, CASP3-Pro_com_ and CASP7-Ser_holo_, CASP7-Ser_com_ respectively. MD simulations for these *in silico* mutants were performed identical to the simulations starting from the original structures.

### Visualization of specificity and flexibility landscapes

For visualization of specificity subpocket-wise cleavage entropies were mapped to the starting coordinates of Caspases 3 and 7, respectively. Likewise, average C_α_ B-factors of subpocket interface residues normalized to the average C_α_ B-factors of all protein residues were mapped to the catalytic cleft of the caspases to allow intuitive visualization. A binding site definition of Yoshimori *et al*. was used to group interface residues into the respective binding sites.^20^ Pictures were generated with Pymol.^59^

## RESULTS

### Substrate specificity of caspases

**Figure 1:**
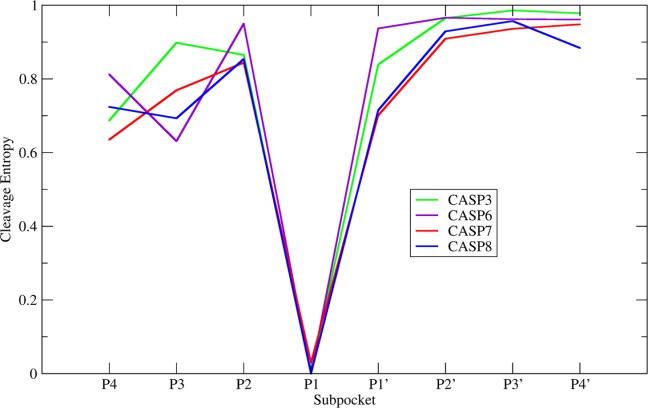
Substrate promiscuity of caspases: Subpocket-wise cleavage entropies of caspases 3, 6, 7, and 8. All members show substrate specificity in the tetrapeptide P-region. Especially the S1 pocket shows stringent specificity for its canonical amino acid substrate Asp. Caspases mainly differ in substrate promiscuity of the P3 and P4 position. Here, Caspase 3 shows higher cleavage entropy and thus less stringent substrate specificity.

Extraction of cleavage information of the apoptosis signaling members of the caspase family from the MEROPS database and quantification of their substrate specificity by cleavage entropy^15^ yielded similar but not identical specificity profiles of individual caspase isoforms (see Fig. 1). All four members Caspases 3 (619 known substrates), 6 (201 substrates), 7 (170 substrates), and 8 (66 substrates) show substrate readout in the P-region. Especially the S1-pocket shows stringent substrate readout with a maximum cleavage entropy of 0.030. The only major differences in cleavage entropy are observed at the P3 substrate position, where Caspase 3 shows rather unspecific binding (*S*_cleavage,P3_ = 0.898), even compared to a cleavage entropy of 0.769 for the close homologue Caspase 7. The same trend holds true for the P4 position, where Caspase 3 binds more diverse substrates (*S*_cleavage,P4_ = 0.687) than Caspase 7 (*S*_cleavage,P4_ = 0.635).

**Figure 2:**
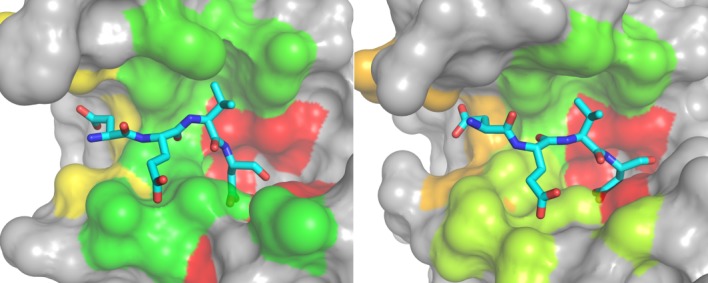
Specificity landscapes of Caspases 3 and 7: Subpocket-wise cleavage entropies were mapped to the binding site of the caspases 3 (left figure) and 7 (right figure) in standard orientation on a color gradient from red (highly specific) over yellow to green (totally unspecific). The catalytic cleft shows a deep hole specifically binding aspartate residues (S1 pocket, right) as well as more accessible and less specific regions (S4–S2, left). The comparison between caspases 3 and 7 shows that the S3 and S4 pocket of Caspase 7 is more specific and hence shifted to red on the color gradient.

Mapping these substrate specificities to the binding sites of Caspases 3 and 7 allows to intuitively visualize patterns of substrate readout (see Fig. 2). The most stringent substrate readout is imposed by the deep S1 pocket binding almost exclusively Asp residues. S2 and S3 pockets are less specific, whereas the S4 pocket again shows pronounced substrate readout, though clearly less specific than the prominent S1 interaction. A comparison of Caspases 3 and 7 shows that Caspase 3 shows more promiscuous substrate binding especially in the S3 and S4 region.

### Flexibility landscapes of caspases

MD simulations were performed to probe binding site flexibility of dimeric Caspases 3 and 7 in holo (CASP3_holo_, CASP7_holo_) and complexed state (CASP3_com_, CASP7_com_). As the active site was engineered from a covalently bound adduct to a nonbonded protein–ligand complex or ligand-free state, a long equilibration time of 50 ns was allowed. Production runs over additional 50 ns yielded stable trajectories for analysis of the dynamics of the catalytic cleft for all simulations. In a comparison of holo and complex simulations, we observed similar conformational ensembles in the respective simulations of both caspases (see Supporting Information Figure S1 for details).

For in-depth analyses of local binding site dynamics, interface residues of the catalytic cleft were grouped to respective subpockets yielding an average local B-factor. Upon complexation with the substrate, we observe a rigidification of the binding site of the binding site indicated by lowered B-factors, thus narrowing the sampled conformational space of the binding site region (see Table1). Relative ordering of B-factors of the individual subsites is not changed upon ligand binding, hence restricting but preserving active site dynamics.

**Table I:** Active Site Dynamics B-Factors of the S4–S1 Region of Caspases 3 and 7 in Holo and Complex Form

Average normalized B-factor	CASP3_holo_	CASP3_com_	CASP7_holo_	CASP7_com_
S4	2.068	1.314	1.221	1.056
S3	2.658	1.623	1.285	1.044
S2	1.055	0.944	0.970	0.984
S1	0.578	0.587	0.447	0.454

The presented values show an average B-factor over two subunits normalized by the average B-factor over the whole protein. Hence a value of 1 shows average flexibility within the protein. Holo proteins are consistently more flexible than ligand-bound complexes. Caspase 3 is found to be more flexible than Caspase 7 in the S3/S4 region.

**Figure 3:**
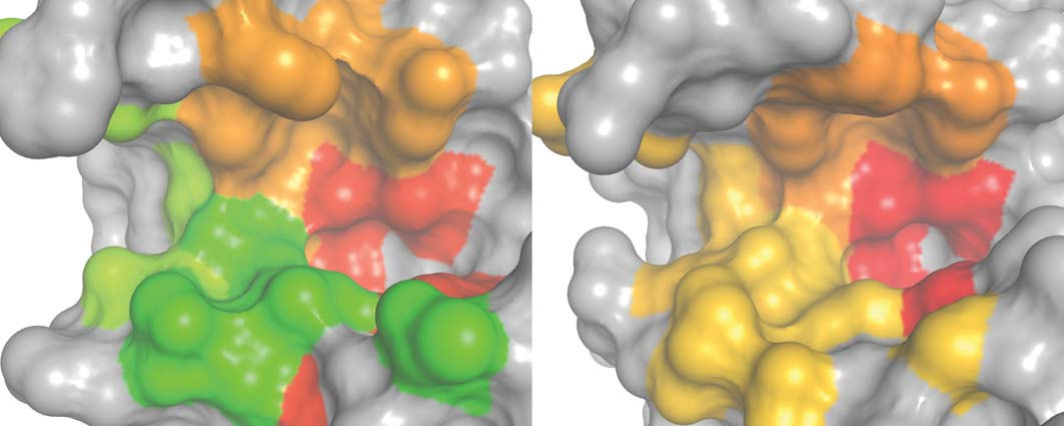
Flexibility landscapes of Caspases 3 and 7: Subpocket-wise average normalized B-factors of holo simulations were mapped to the binding site of caspases 3 (left figure) and 7 (right figure) in standard orientation on a color gradient from red (rigid) over yellow to green (flexible). The S1 pocket (right) is shown to be very rigid, whereas the S3 and S4 region (left) shows elevated dynamics in case of Caspase 3, whereas these pockets remain relatively rigid for Caspase 7.

To reveal connections between binding site flexibility and specificity, we correlated subpocket-wise cleavage entropies (see Fig. 2) from database analyses to B-factor profiles of MD simulations (see Table1). The S1-pocket was found to be consistently the most specific and most rigid binding pocket of Caspases 3 and 7 in holo as well as complexed state (for a mapping to the binding site region, see Fig. 3). The apolar S2-pocket of caspases is found to oppose the observed positive correlation between specificity and local rigidity. However, S3 and S4 again follow initially observed trends. These subpockets were found to exhibit less pronounced substrate readout in data mining and were found to more flexible in MD simulations. The overall Spearman rank correlation coefficients for specificity and rigidity of the four simulations were found in the range of 0.2–0.8 over S1–S4 pockets.

A comparison between active site flexibility of Caspases 3 and 7 shows conserved flexibility patterns in the S1 and S2 region. Nevertheless, S3 and S4 show a clear distinction between the two effector caspases: Caspase 3 has more flexible outer pockets than Caspase 7. Again, this finding holds true for MD simulations of the holo protease as well as the complexed form.

### *In silico* exchange mutations in the S3 pocket

A structural superposition of Caspases 3 and 7 highlights a major difference in the S3 pocket of the proteases: Caspase 3 contains a serine residue (Ser-343) in this subpocket that is replaced by a proline (Pro-235) in Caspase 7. The spatial restrictions imposed on local backbone movement by this proline residue in Caspase 7 could explain the observed subpocket rigidification. Hence, an exchange mutation in the S3 pocket of the superimposed Ser-343 in Caspase 3 and Pro235 in Caspase 7 was performed. Subsequently, MD simulations were performed as for the native proteins in holo and complexed states (CASP3-Pro_holo_, CASP7-Ser_holo_, CASP3-Pro_com_, and CASP7-Ser_com_). Upon *in silico* mutation we observe preserved hydrogen bonding patterns and subpocket geometries, proving plausibility of our simulations (see also “Analysis of protein–ligand interactions” section).

**Figure 4:**
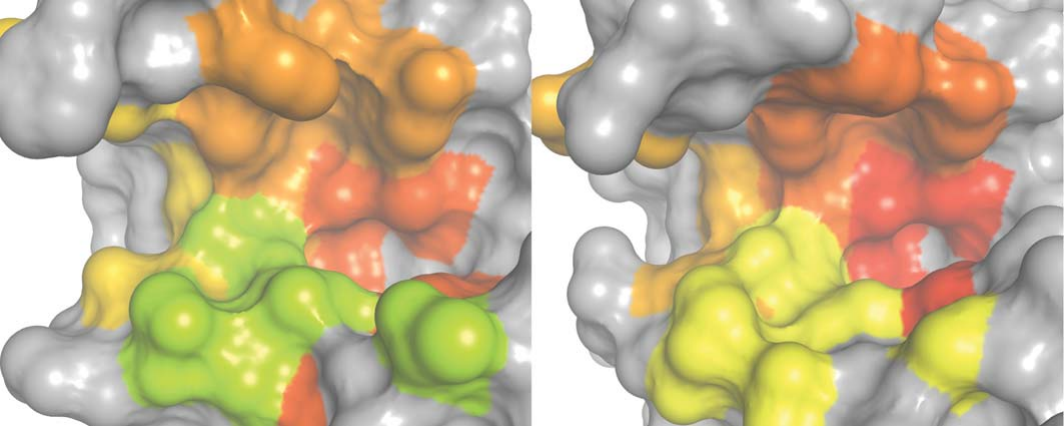
Flexibility landscapes of *in silico* mutants Caspase-3-Pro and Caspase-7-Ser: Subpocket-wise average normalized B-factors of holo simulations were mapped to the binding site of Caspases 3 (left figure) and 7 (right figure) in standard orientation on a color gradient from red (rigid) over yellow to green (flexible). Upon *in silico* mutation general binding site characteristics are preserved. Still, the S3 subpocket (bottom left), where the mutation was applied, is rigidified in Caspase 3 via a Pro → Ser mutation. The same binding site region is mobilized by a Ser → Pro mutation in Caspase 7.

Again, B-factors of residues in protease subpockets were grouped to yield an average measure for local dynamics in the mutant simulations (see Table2). Mapping differences in dynamics of the MD simulations (see Fig. 4) allows to analyze the impact of the S3 mutations on local binding flexibility: The proline residue in Caspase 3 consistently rigidifies the S3 and S4 region indicated by a reduced average B-factor, whereas the serine residue in Caspase 7 mobilizes the S3 pocket. Flexibility of the S2 pocket remains unaffected by the *in silico* mutations, whereas S1 is mobilized in three of four simulations. We attribute this considerable relative change to the small absolute B-factors in the very rigid S1 pocket of caspases.

**Table II:** Active Site Dynamics B-Factors of the S4–S1 Region of Caspase-3-Ser and Caspase-7-Pro Mutants in Holo and Complex Form Each

Average normalized B-factor	CASP3-Pro_holo_	CASP3-Pro_com_	CASP7-Ser_holo_	CASP7-Ser_com_
S4	1.348 (−35%)	1.277 (−3%)	1.182 (−3%)	1.030 (−2%)
S3	1.947 (−27%)	1.499 (−8%)	1.577 (+23%)	1.107 (+6%)
S2	1.037 (−2%)	1.034 (+10%)	0.827 (−15%)	0.964 (−2%)
S1	0.750 (+30%)	0.719 (+22%)	0.578 (+29%)	0.427 (−6%)

The presented B-factors are averaged over two subunits and normalized to the average B-factor within the protein. Therefore, a relative B-factor of 1 indicates a region of average flexibility within the protein. Differences to the native proteins are indicated in brackets. As in native state, protein active sites are more flexible in both ligand-free simulations. Compared to simulations of the native protein, the S3 pocket of Caspase 3 is rigidized by introduction of a proline residue, whereas S3 of Caspase 7 is mobilized via mutation of a proline to a serine.

### Analysis of protein–ligand interactions

Findings from B-factors are further confirmed by additional analyses of the binding site geometries. Upon *in silico* mutations the distance of the S3-bound glutamate of the inhibitor to the proline/serine residue is preserved (5.6 Å and 5.3 Å, respectively, see Table3). In contrast to the average distance, standard deviations (SDs) of this distance highlight the impact of this mutation on local dynamics: The highly dynamic S3-pocket CASP3_com_ (SD = 19.84%) is rigidified via introduction of the proline in CASP3-Pro_com_ (SD = 13.25%). This standard deviation is almost as low as measured for CASP7_com_ (SD = 11.71%). Upon Pro → Ser mutation dynamics are elevated to a SD of 17.17% in the simulation of CASP7-Ser_com_.

**Table III:** Protein–Substrate Distances in the S3 Pocket

	CASP3_com_	CASP7_com_	CASP3-Pro_com_	CASP7-Ser_com_
Average distance (Å)	5.62	5.31	5.65	5.33
Standard deviation (%)	19.84	11.71	13.25	17.17

Averages and standard deviations over 50 ns and two subunits of the distances between the S3-bound glutamate and the C_β_, as last comparable atom, of Pro/Ser-235/343 of caspases and their mutants are shown. We observe a tighter packing within S3 of Caspase 7 leading to a more stringent substrate readout. Distances are preserved upon *in silico* exchange mutation of proline and serine, but standard deviations as measure of flexibility are affected. Binding site characteristics of caspases 3 and 7 are inverted by *in silico* mutations: Caspase 3 is rigidized, whereas Caspase 7 is mobilized.

An analysis of protein–ligand hydrogen bonding occupancy over the simulation time elucidates the impact of binding site rigidity on protein–ligand interactions (see Table4). Whereas in native simulations Caspase 3 forms on average 6.87 hydrogen bonds to the inhibitor peptide, the rigid Caspase 7 forms 8.28. This difference can mostly be attributed to an increase of hydrogen bonding in the S3 and S4 pockets. In the simulation of the CASP3-Pro mutant, S3/P3 interactions are increased leading to a total of 7.85 hydrogen bonds in the rigidified CASP3-Pro_com_. The introduction of the serine in Caspase 7 does not alter hydrogen bonding patterns, preserving subpocket contributions as well as a total average of 8.23 hydrogen bonds in CASP7-Ser_com_.

**Table IV:** Protein–Substrate Hydrogen Bonding in the Binding Site Region P4–P1

	CASP3_com_	CASP7_com_	CASP3-Pro_com_	CASP7-Ser_com_
S4–P4	1.37	1.99	1.38	2.03
S3–P3	2.46	2.99	3.34	3.03
S2–P2	0.00	0.00	0.00	0.00
S1–P1	3.04	3.30	3.13	3.17
Total	6.87	8.28	7.85	8.23

Occupancies of protein–substrate hydrogen bonds are averaged over 50 ns and two subunits. P2–S2 interactions are unspecific due to the complete absence of hydrogen bonds in the hydrophobic S2 pocket of caspases. A striking increase of hydrogen bonding occupancy within the S3 pocket of Caspase 3 is observed upon introduction of a proline residue. This local increase is also reflected in the total hydrogen bond count.

## DISCUSSION

Calculation of subpocket-wise cleavage entropies was found useful to rationalize subtle differences in substrate specificities of effector Caspases 3 and 7. Consistent with qualitative data from the literature, we found Caspase 3 to exhibit less stringent substrate readout. Especially, subpockets S3 and S4 were found to differ in substrate readout, allowing Caspase 3 to cleave more diverse substrates than Caspase 7. As a superposition of the two proteases did not yield a structural explanation for these distinct substrate promiscuities, we performed MD simulations to assess the proteins' conformational space.

After a long equilibration phase of 50 ns stable trajectories for Caspases 3 and 7 in holo and complexed states over another 50 ns were generated. Grouping of interface residues into respective subpockets yielded a flexibility profile in the active site of the caspases based on B-factors. A consistently positive Spearman ranking correlation coefficient between local flexibility and substrate promiscuity over 4 subpockets in independent four simulations highlighted the importance of conformational dynamics for the investigated systems. On a structural level, this intrinsic link might be attributed to the presence of long flexible flaps surrounding the binding pocket of caspases.

The narrower conformational space of the more rigid Caspase 7 provides a smaller set of possible receptor conformations for the substrate peptide following a binding model of conformational selection.^8^,^9^ This interpretation is plausible due to the stability of holo simulations started from ligand-bound coordinates and the very similar structure of free and ligand-bound state.^41^ The lower diversity of receptor conformations directly implies restrictive substrate readout, as a smaller amount of peptide configurations can be bound.

The general importance of protease dynamics in substrate readout has been identified years ago, especially the crucial role of surface loops.^69^ Local dynamics as mechanism of substrate readout in addition to direct protein–ligand interactions could explain observed problems in exchanging substrate specificity between members of the chymotrypsin fold.^70^,^71^ For this fold family of serine proteases, a mechanism of conformational selection on the nanosecond timescale has been proven experimentally.^72^ Dynamics were later shown to balance selectivity and promiscuity in the fold member thrombin.^73^,^74^ Further proteases, where a inherent correlation between substrate readout and dynamics has been observed include alytic protease,^75^,^76^ HIV protease,^77^ members of the subtilisin fold,^78^ and snake venom metalloproteases.^79^ Furthermore, conserved active site dynamics in the protease superfamily imply importance of considering flexibility aspects in the understanding of proteolytic enzymes.^80^

Still, direct interactions between protease and substrate peptide play a key role in sequence specificity and promiscuity.^81^ Three of four investigated caspase subpockets preferably bind negatively charged amino acids, only the S2 subpocket prefers hydrophobic residues. Hence, nonspecific ligand binding to the S2 pocket can be attributed to the lack of direct molecular interactions supporting substrate readout in other subpockets (cf. also Table3). Removing the S2 pocket from analyses consistently improves the observed Spearman rank correlation coefficients between flexibility and cleavage entropy for the four simulations Caspases 3 and 7 in holo and complexed state. Therefore, nonspecific substrate readout of the rigid but unspecific S2 subpockets of Caspases 3 and 7 is governed by the lack of direct interactions in contrast to other pockets, where dynamics play a key role. The S1 pocket remains conserved as rigid and highly specific part of the binding site of the caspases. Differences in specificity are relevant in the S3 and S4 pockets, where the broader substrate recognition of Caspase 3 is allowed via a broader conformational space. We found Pro-235 as key regulator of local rigidity in Caspase 7 that is not present in Caspase 3 showing Ser-343 as coinciding residue upon superposition.

To confirm the importance of Pro-235 we performed *in silico* exchange mutations and additional MD simulations using identical settings as for simulations of the native structures. Although a serine residue was artificially introduced in the S3-engineered system CASP7-Ser, this serine residue does not form additional hydrogen bonds to the substrate. Rather, we observe a mobilization of the S3 subpocket leading to a CASP3-like protease via the introduction of the serine residue. In the vice versa mutation the system CASP3-Pro is rigidified as expected by the additional constraint on backbone dynamics implied by the introduced proline.

Sufficient information on cleaved substrates is available for two further apoptotic members of the caspase fold: Caspases 6 and 8. These two enzymes do not share sequence similarity to a degree as high as Caspases 3 and 7. Still, a major feature in the S3 pocket can be observed: Caspases 6 and 8 both contain a proline residue in this subpocket.^20^ When retrospectively analyzing cleavage entropy profiles (see Fig. 1), one can classify caspases into two groups showing different substrate readout in the S3. Caspase 3 exhibits unspecific ligand binding in the S3 (*S* = 0.898), whereas Caspases 6, 7, and 8 share similar substrate readout (*S* = 0.631–0.769). Interestingly, this splitting into two groups can also be observed on the sequence and structure level: Caspase 3 is the only investigated caspase lacking a proline residue in S3. Thus, we speculate that findings for Caspases 3 and 7 could be generalized for the whole caspase family. Unfortunately, a detailed computational analysis of additional fold members is not in reach, as structural information of these proteins bound to the same substrate is currently not available.

This observed interchange of binding site dynamics in caspases naturally raises the question, if also substrate specificity and promiscuity could be interchanged via a transformation of active site flexibility. Hence, we propose to experimentally express the systems CASP7-Ser and CASP3-Pro and assess impact of the mutations on structure, dynamics and substrate binding. If CASP7-Ser indeed exhibits more promiscuous behavior than native CASP7, the proposed conformational selection mechanism governing substrate readout could be strengthened. An experimental confirmation could hence guide the way toward the rational design of proteases with desired substrate specificity, which should than be based more on an ensemble perspective of protease conformations rather than a static view. MD simulations could lead the way to a new era in the understanding and design of protein function, thereby complementing static structural studies.

## CONCLUSION

In conclusion, we show that dynamics play a key role in substrate readout of caspases. Besides direct interactions of protease and substrate, dynamics determine the available conformational space of the proteases. Following a mechanism of conformational selection the availability of diverse receptor conformations allows to bind more diverse substrates and hence leads to promiscuous binding sites. In this study we show that a proline residue in the S3 of the more specific Caspase 7 introduces rigidity into this subpocket in contrast to Caspase 3 with a serine at the same position. Despite the absence of direct interactions with the substrate, this proline narrows the conformational space of the subpocket sufficiently to achieve stringent substrate readout. This key role of the proline residue is not evident from inspection of a single static structure, but becomes available by sampling of the conformational space via MD simulations.

Following the assumption, that active site dynamics generally govern specificity of protein–protein interfaces one could argue that an ensemble perspective of these biomolecular interactions is necessary to understand their specificity. Hence, we propose to theoretically and experimentally study dynamics of a broad range of protein–protein interfaces to assess their impact on specificity. On the other hand, an expression of the proposed model systems CASP3-Pro as well as CASP7-Ser and a study of their respective substrate specificity would be highly interesting to experimentally proof trends proposed by this study.
