# Supplementary material for: Specificity of a protein–protein interface: Local dynamics direct substrate recognition of effector caspases
Source: Proteins. 2014 Oct 19;82(4):546–55. doi: 10.1002/prot.24417 (PMC4282588; doi:10.1002/prot.24417)
Supplement: Supplementary file 1 [file prot0082-0546-SD1.pdf]

PLOS Computational Biology

Substrate-Driven Mapping of the Degradome by Comparison of Sequence Logos

--Manuscript Draft--

Manuscript Number:	
Full Title:	Substrate-Driven Mapping of the Degradome by Comparison of Sequence Logos
Short Title:	Substrate-Driven Mapping of the Degradome
Article Type:	Research Article
Keywords:	Degradome; Sequence Logos; Protease Specificity Tree; Substrate Recognition; Protease Similarity Metric
Corresponding Author:	Klaus R Liedl<br>University of Innsbruck<br>Innsbruck, AUSTRIA
Corresponding Author Secondary Information:	
Corresponding Author's Institution:	University of Innsbruck
Corresponding Author's Secondary Institution:	
First Author:	Julian E Fuchs
First Author Secondary Information:	
Order of Authors:	Julian E Fuchs
	Susanne von Grafenstein
	Roland G Huber
	Christian Kramer
	Klaus R Liedl
Order of Authors Secondary Information:	
Abstract:	Sequence logos are frequently used to illustrate substrate preferences and specificity of proteases. Here, we employed the compiled substrates of the MEROPS database to introduce a novel metric for comparison of protease substrate preferences. The constructed similarity matrix of 62 proteases can be used to intuitively visualize similarities in protease substrate readout via principal component analysis and construction of protease specificity trees. Since our new metric is solely based on substrate data, we can engraft the protease tree including proteolytic enzymes of different evolutionary origin. Interestingly, our analyses show pronounced overlaps in substrate recognition not only between proteases closely related on sequence basis but also between proteolytic enzymes of different evolutionary origin and catalytic type. To illustrate the applicability of our approach we analyze the distribution of targets of small molecules from the ChEMBL database in our substrate-based protease specificity trees. We observe a striking clustering of annotated targets in tree branches even though these grouped targets do not necessarily share similarity on protein sequence level. This highlights the value and applicability of knowledge acquired from peptide substrates in drug design of small molecules, e.g., for the prediction of off-target effects or drug repurposing. Consequently, our similarity metric allows to map the degradome and its associated drug target network via comparison of known substrate peptides. The substrate-driven view of protein-protein interfaces is not limited to the field of proteases but can be applied to any target class where a sufficient amount of known substrate data is available.
Suggested Reviewers:	Neil D Rawlings<br>Sanger Institute, Cambridge, UK<br>ndr@sanger.ac.uk<br>Protease Expert, Curator of MEROPS Database

Powered by Editorial Manager® and ProduXion Manager® from Aries Systems Corporation

	Oliver Schilling<br>Institut für Molekulare Medizin und Zellforschung, Universität Freiburg, Germany<br>oliver.schilling@mol-med.uni-freiburg.de<br>Protease and Degradomics Expert
	Chris M Overall<br>Centre for Blood Research, University of British Columbia, Vancouver, Canada<br>chris.overall@ubc.ca<br>Protease and Degradomics Expert
	Carlos Lopez-Otin<br>Department of Biochemistry and Molecular Biology, Universidad de Oviedo<br>clo@uniovi.es<br>Protease Structure and Protease Genomics Expert
**Opposed Reviewers:**	

Powered by Editorial Manager® and ProduXion Manager® from Aries Systems Corporation

Univ.-Prof. DDr. Klaus R. Liedl
Institut für Allgemeine, Anorganische und Theoretische Chemie
Universität Innsbruck, Innrain 80/82, A-6020 Innsbruck, Austria
Tel.: +43/512/507/57100, Fax: +43/512/507/57199, E-Mail: Klaus.Liedl@uibk.ac.at

Prof. Ruth Nussinov
School of Medicine
Tel Aviv University
Israel

Innsbruck, June 18th, 2013

Dear Editor,

enclosed please find the manuscript of our paper "Substrate-Driven Mapping of the Degradome by Comparison of Sequence Logos".

Herein we present a novel method for comparison of proteases solely based on their substrate specificity. We show that our new metric helps to intuitively visualize similarities in protease substrate recognition. As our new method is not based on protease sequences, it allows to compare non-homologous proteases in respect to their substrate binding preferences.

In a first application of our protease specificity trees we show how knowledge from peptide substrates can be directly transferred to small molecule inhibitors. Our substrate-driven protease trees can hence be exploited to predict off-target effects and to repurpose drugs.

As our metric for similarity can be applied for any sort of sequence motif or multiple sequence alignment, we think that the presented approach is of general interest for the readers of *PLOS Computational Biology*. Furthermore, the substrate-driven analysis could be expanded to kinases or any sort of protein-protein interfaces, thus allowing to highlight similarities even in absence of any homology.

We kindly ask you to consider our manuscript for publication in *PLOS Computational Biology* as a research article.

Yours sincerely

Klaus Liedl

*Manuscript
Click here to download Manuscript: manuscript.pdf

Substrate-Driven Mapping of the Degradome by Comparison of Sequence Logos

Authors:
Julian E. Fuchs†, Susanne von Grafenstein†, Roland G. Huber†, Christian Kramer†, Klaus R. Liedl†*

† Institute of General, Inorganic and Theoretical Chemistry, and Center for Molecular Biosciences Innsbruck (CMBI), University of Innsbruck, Innrain 80/82, A-6020 Innsbruck, Austria

*Corresponding Author
E-mail: klaus.liedl@uibk.ac.at

Abstract

Sequence logos are frequently used to illustrate substrate preferences and specificity of proteases. Here, we employed the compiled substrates of the MEROPS database to introduce a novel metric for comparison of protease substrate preferences. The constructed similarity matrix of 62 proteases can be used to intuitively visualize similarities in protease substrate readout via principal component analysis and construction of protease specificity trees. Since our new metric is solely based on substrate data, we can engraft the protease tree including proteolytic enzymes of different evolutionary origin. Interestingly, our analyses show pronounced overlaps in substrate recognition not only between proteases closely related on sequence basis but also between proteolytic enzymes of different evolutionary origin and catalytic type.

To illustrate the applicability of our approach we analyze the distribution of targets of small molecules from the ChEMBL database in our substrate-based protease specificity trees. We observe a striking clustering of annotated targets in tree branches even though these grouped targets do not necessarily share similarity on protein sequence level. This highlights the value and applicability of knowledge acquired from peptide substrates in drug design of small molecules, e.g., for the prediction of off-target effects or drug repurposing. Consequently, our similarity metric allows to map the degradome and its associated drug target network via comparison of known substrate peptides. The substrate-driven view of protein-protein interfaces is not limited to the field of proteases but can be applied to any target class where a sufficient amount of known substrate data is available.

Authors Summary

We present a novel approach to intuitively map the degradome, the set of proteolytic enzymes, based on their substrates rather than on the protease sequences. Information stored in cleavage site sequence logos is extracted and transferred into a metric for similarity in protease substrate recognition. By capturing similarity in substrate readout, we inherently focus on the biomolecular recognition process between protease and substrate. Furthermore, we are able to include proteases of different evolutionary origin into our analysis, because no assumption on homology has to made.

In a second step, we show how knowledge from peptide substrates can directly be transferred into small molecule recognition. By mining protease inhibition data in the ChEMBL database we show, how our substrate-driven protease specificity trees group known targets of protease inhibitors. Thus, our substrate-based maps of the degradome can be utilized in the prediction of off-target effects or drug repurposing. As our approach is not limited to the protease universe,

our similarity metric can be expanded to any kind of protein-protein interface given sufficient substrate data.

Introduction

The degradome, the complete set of proteolytic enzymes [1], comprises more than 500 proteases in humans, where every single one is linked to a unique cleavage pattern [2]. Although they all share the same catalytic principle, which is the hydrolytic cleavage of a peptide bond [3 substrate spectra range from the specific degradation of single peptides to promiscuous non-specific degradation of multiple substrates [4]. Therefore, proteases can execute a wide range of biological functions, from specific signaling tasks to unspecific digestion of nutrition proteins [5]. Proteases initiate, modulate and terminate a wide range of fundamental cellular functions [6], making them attractive targets for drug design [7].

Substrate specificity of proteases is determined via molecular interactions at the protein-protein interface of the substrate with the proteolytic enzyme. Specificity subpockets necessary for recognition of substrates as well as substrate positions are numbered according to the convention of Schechter and Berger [8]: Peptide amino acids P are indexed with position 1 around the scissile bond, with P1' being oriented towards the C-terminal. Indices are incrementally increased for subpockets farther away from the bond about to be cleaved. Protease subpockets binding the substrates are numbered Sn-Sn', ensuring consistent indices for substrate and enzyme pockets interacting directly. The peptide substrate is typically locked in a canonical beta conformation [9] spanning several subpockets flanking the catalytic center explaining specificity for the substrate sequence [10,11].

Known proteases cover several types of catalytic machineries including aspartic, cysteine, metallo, serine and threonine proteases according to the MEROPS database [12]. Still, some of these protease groups include non-homologous members allowing further subdivision into clans and families. Serine proteases may be subdivided into homologous clans such as the chymotrypsin fold, the subtilisin fold, or the carboxypeptidase Y fold. This inherent complexity of proteolytic systems [13,14] is tackled by a broad range of research activities to profile protease specificity [15]. Established methods for substrate profiling include chromatography-based methods [16], phage display [17], usage of substrate libraries [18,19] and fluorogenic substrates [20] as well as N-terminal labeling techniques [21]. Still, inherent similarities in protease substrate readout have by now only been examined qualitatively (e.g. [22]).

Apart from a solid classification of known proteases, MEROPS contains a collection of known substrates [23] even exceeding 10000 known substrates in case of trypsin 1. This substrate sequence data is frequently depicted as sequence logos [24] or heat maps [25] to highlight individual substrate preferences of proteases. Recently, substrate information from MEROPS has been successfully employed in the prediction of protease cleavage sites using machine learning techniques [26] or the calculation of cleavage entropy, a quantitative measure of substrate promiscuity [4].

In our current study, peptide substrate data set from the MEROPS database forms the basis of an approach to map the complex world of proteases into intuitively accessible diagrams by highlighting similarities in substrate readout between individual proteases. An extraction of known protease inhibitors from the ChEMBL database [27] shows, how knowledge from peptide substrates can be directly transferred into predictions on small molecules. Overlaps in cleaved peptides correlate with binding of similar small molecules, thus indicating overlaps in the chemical space covered. This observation renders our approach promising for the prediction of off-target effects or general chemogenomic approaches in drug discovery.

Methods

- Extraction and Processing of Substrate Data

Data on known substrates were downloaded from the MEROPS database [12] containing the largest collection of substrate sequences when compared to other online resources as CutDB [28] or Proteolysis MAP [29]. All proteases with at least 100 annotated substrates were selected for further analysis, forming an initial set of 65 proteases. Three aminopeptidases were discarded, as half of their binding site remains unoccupied, yielding a final set of 62 proteases (see Supporting Table S1 for a detailed list). Sequence logos depicting respective substrate preferences were generated with WebLogo [30]. For each protease, a sequence matrix covering eight positions S4 to S4' based on the frequency of each of the 20 natural amino acids was generated. Residue frequencies were normalized to their natural abundance [31] to ensure a proper reflection of protease substrate preferences.

- Calculation of Protease Substrate Similarities

For each subpocket we extracted a vector of length 20 containing the respective amino acid frequencies at that position from the sequence matrix. In order to facilitate a comparison of the whole binding frame or regions within, respective vectors for subpockets were combined and normalized to yield a substrate vector v of length one and dimension 160 for the eight binding pockets. Apparently, comparison of smaller binding site regions results in lower dimensional vector spaces. Similarities between vectors were calculated as scalar products (dot products). The scalar projection of one normalized vector on another yields an overlap of 1 for identical vectors and an overlap of 0 for orthogonal vectors. Thus, such a metric is perfectly suitable to quantify similarities s of amino acid distributions encoded in the vectors v (see Formula 1 and Figure 1 for a summary).

$$s=\vec{v}_1\cdot\vec{v}_2=\left(p_{S4,Ala}\,p_{S4,Arg}\cdots p_{S4,Val}\,p_{S3,Ala}\cdots p_{S4',Val}\right)_1\cdot\left(p_{S4,Ala}\,p_{S4,Arg}\cdots p_{S4,Val}\,p_{S3,Ala}\cdots p_{S4',Val}\right)_2$$

Formula 1: Calculation of protease similarities s based on substrate vectors v_1, v_2 containing amino acid frequencies p at each subpocket of the binding site

A complete pairwise comparison of all 62 cleavage site sequence logos stored as vectors yield a symmetric matrix of dimension 62 with values of 1 for the comparison of identical substrate vectors in the main diagonal. A distance matrix was created by subtraction of all elements of the similarity matrix from 1. Hence, a pairwise distance of 0 represents identical substrate recognition, whereas 1 depicts maximal distance in protease space. The resulting distance matrix stores differences in substrate recognition of all 62 proteases in the test set.

- Analysis of the Protease Distance Matrix

The distance matrix of 62 protease substrate recognition patterns was diagonalized using SciPy [32]. Principal components of the matrix were extracted as eigenvectors in protease space. Corresponding eigenvalues normalized to the sum of all eigenvectors depict the individual contribution of the eigenvector to the total variance in the data set. Principal components were sorted according to their contribution and depicted as loadings plots. Subpocket-wise cleavage entropies and total

cleavage entropies were calculated as described earlier [4].

- Construction of Substrate-Driven Protease Specificity Trees

Apart from directly analyzing the protease distance matrix via principal component analysis, we visualized similarities in protease substrate recognition as dendrograms. We used fkitsch from the EMBOSS server [33] employing a Fitch Margoliash method [34] for tree construction. 100 random starts were performed to ensure robustness of constructed similarity trees. Interactive Tree of Life (iTOL) was used to visualize the constructed substrate-driven protease specificity trees [35]. Although we think that the statistical term "selectivity" would better fit our presented analysis, we stick to the long-established phrase "protease specificity".

- Mapping of Ligand Data

We used the ChEMBL database version 16 [27] as resource for small molecule bioactivity data. ChEMBL lists 1.5 million compounds with more than 11 million associated bioactivities. We extracted all 426 protease targets, associated selectivity groups as well as annotated ligands. A list of matched MEROPS and ChEMBL identifiers is provided in Supporting Table S1. We discarded covalent inhibitors from our analysis and mapped the remaining bioactivities to our protease specificity trees. We did not employ a stringent activity cutoff but rather preserved all annotated target affinities as positives to provide a comprehensive picture of protease-ligand recognition. Only empty fields or zero percent inhibition annotations were discarded.

Results

- Quantification of Similarity in Protease Substrate Readout

Data mining in the MEROPS database showed the increasing importance and promise of knowledge-based approaches in recent years, as for example described by Ekins et al [36]. Within 18 months, the set of proteases with more than 100 cleavage sites annotated in MEROPS increased from 47 [4] to currently 65. After discarding three aminopeptidases, the set of 62 proteases spans the four major catalytic types of proteases: serine, metallo, cysteine, and aspartic proteases (see Supporting Table S1 for details). No member of glutamic, threonine and asparagine proteases qualified for inclusion into our study due to insufficient substrate data for all their members.

Our presented approach yields quantitative distance values between known protease substrate preferences. A value of 0 represents identical substrate readout, whereas 1 shows an orthogonal cleavage pattern in all subpockets investigated. Calculated distance values within the set of proteases span a wide range. Distances in substrate readout range from 0.003 to 0.79 when calculated over the whole range of eight subpockets flanking the cleavage site (S4-S4'). This finding highlights the diversity of substrate recognition among known proteases.

A group with nearly identical substrate recognition are the proprotein convertases of MEROPS subfamily S8B, which uniformly cleave after two basic residues [37], reflected in a distance lower than 0.1 between all members except kexin. In contrast to all other members, kexin does not recognize arginine residues in the S4 pocket, hence leading to higher distance values up to 0.29. A further group with highly similar substrate recognition are the apoptotic signalling caspases 3 and 7 [38] with a distance lower than 0.05. Although both share DEVD as ideal substrate in the non-prime region [39], they were

found to have functionally different effects [40]. Further groups that recognize highly similar substrates comprise thrombin and plasmin cleaving after basic residues [20,41], as well as unspecific matrix metallo proteases showing a high degree of overlap between substrates [42,43]. Except cathepsins K, L, B, S, H and V, these groups of similar substrate recognition nicely coincide with annotated protease selectivity groups within ChEMBL.

The cell signaling peptidases neurolysin and thimet oligopeptidase were found to form a group with similar substrate readout (distance = 0.033) which is very distinct to all others protease within the set (all distances > 0.45). Both peptidases hydrolyze a narrow spectrum of intracellular oligopeptides [44,45] whilst sequence readout is spanning over the whole binding site region from S4 to S4' [4]. The largest distance within the protease set is found between KPC2type peptidase of *Caenorhabditis elegans*, a subtilisin-like proprotein convertase, that specifically cleaves a group of neuropeptides [46], and the unspecific matrix metallo protease 13 [47]. Intuitively, distances between unspecific proteases are smaller, e.g., the distance between substrate recognition of both highly promiscuous thermolysin and chymotrypsin is found to be lower than 0.22.

- Principal Component Analysis of the Protease Similarity Matrix

Compiling all pairwise protease substrate similarities yields a symmetric matrix representing distances in substrate readout of the 62 investigated proteases. Principal component analysis of this matrix reveals that the first principal component, depicting a linear combination of protease substrate recognition patterns, is sufficient to cover 50 percent of variance within the data set. Second and third axis contribute 8.9 and 5.9 percent respectively, while the seventh principal component shows the last contribution exceeding 2 percent. These first seven principal components cover more than 77 percent of total variance in the data set and thus represent the main features in protease substrate recognition.

Both first and second principal component (PC1, PC2) strongly correlate with substrate promiscuity measured as total cleavage entropy [4]. Pearson's linear correlation coefficient r for these two axes is 0.87 and 0.79 respectively, indicating a pronounced positive linear correlation. While PC1 shows a strong correlation over the whole binding site region S4-S4', PC2 mainly contains information on substrate specificity in the S4-S1 region. PC2 outnumbers PC1 especially in terms of S1 readout (r=0.72 for PC2 versus r=0.42 for PC1). Hence, the scatter plot of PC1 versus PC2 shows a separation of specific and unspecific proteases as well as via PC2 a separation of serine proteases specifically recognizing positively charged amino acids in the P1 position (see Figure 2a, 2b).

The third principal component (PC3) does not correlate with overall substrate promiscuity but rather with a single substrate position P3' (correlation to subpocket-wise cleavage entropy for P3': r=0.73). Several matrix metallo proteases are known to show amino acid preferences at this position besides the S1' pocket, being the main carrier of substrate specificity in matrix metallo proteases [48]. For example matrix metallo protease 13 is known to preferably cleave peptides having a small residue as glycine or alanine at position P3' [49]. As a consequence, PC3 separates metallo proteases. Still, completely unspecific matrix metallo proteases, as for example thermolysin, are not separated from other proteases via PC3 (see Figure 2c, 2d).

Further principal components rather represent single amino acid preferences at specific positions than general substrate promiscuity. The sixth principal component (PC6) separates aspartic proteases from other catalytic types, as several of them show a preference for apolar residues in P1 position. Therefore, a scatter plot of PC3 versus PC6 nicely clusters the different

catalytic types present in the test set of 62 proteases. Necepsin 1 of *Caenorhabditis elegans* is the only aspartic protease not well separated from other catalytic types. For this particular protease involved in neurodegeneration [50] no stringent substrate criteria are known [51].

- Regrafting the Protease Similarity Tree

The distance matrix of proteases investigated via principal component analysis was also employed to construct a similarity tree based on protease substrate recognition over the whole binding site S4-S4'. Tree construction was found to yield a consistent result at a minimum of 100 random starts with a standard error of seventeen percent on distance reproduction for the tree over the whole binding site.

In contrast to evolutionary trees based on protein sequences or domains (e.g. [52, 53, 54, 4]), similarity trees based on substrate readout allow to compare enzymes of different evolutionary origin because no assumption on homology has to be made. Hence, individual evolutionary trees of proteases are merged to yield a complete picture of diversity in substrate readout of proteases (see Figure 3). Even though no protease sequence information was used in tree construction, information on evolutionary subgroups of proteases is recovered from substrate-driven protease specificity trees. Homologues thimet oligopeptidase and neurolysin are grouped in a separate branch distinct in degradome space from all other members, as both of them cleave oligopeptides with substrate readout over the whole binding site region [45, 4]. A second branch is formed by the subfamily S8B around the subtilases kexin and furin. Non-homologous kallikrein-related peptidase 4 is added to the branch, though being overall more unspecific. Still, it shares the main features of substrate readout: peptides containing positively charged residues at P1 [55] as well as arginine-containing substrates at P4 are preferred (see also Supporting Table S1).

In general, proteases are grouped with respect to substrate promiscuity as measured by total cleavage entropy [4]. The main branch of the protease specificity tree first splits off caspases and granzyme B sharing a preference for aspartate residues at P1, although evolutionary not related and not even sharing the catalytic type. Caspases form a separate fold of cysteine proteases C14 [56], whereas granzymes are members of the chymotrypsin fold of serine proteases S1 [57]. After splitting off several singletons with unique substrate readout, residual proteases form a branch of unspecific matrix metallo proteases M10 as well as the digestive enzymes within the pepsin family A1. Overall, the large branch comprising most proteases spans from individual specific proteases to completely unspecific enzymes.

Apart from a comparison over the whole binding site region, similar analyses were performed for regions of interest within. An analog protease specificity tree was constructed only based on substrate data of the non-prime region S4-S1 (see Figure 4). Similar grouping of proteases was obtained as compared to the protease specificity tree over the whole binding site region. This highlights the importance of interactions within the non-prime region for specific protease substrate recognition. By narrowing the region of interest, catalytic types of proteases as well as evolutionary families are clustering more and more, still preserving the overall trend to group specific as well as unspecific proteases.

When narrowing down the substrate positions analysed to amino acids at P1, the readout at this particular subpocket can be investigated in detail (see Figure 5). The degradome again splits into three main branches in the protease specificity tree. First, proteases recognizing aspartate residues at P1 such as caspases and granzyme B are split off. Second, proteases cleaving after positively charged residues, as for example trypsin [20], are separated. This branch shows an internal

branching pattern according to the preference of arginine over lysine or vice versa. The third branch splits off several proteases showing unique substrate preferences: elastase preferring hydrophobic residues [58], glutamyl peptidase I specifically cleaving after glutamate residues [59] as well as neurolysin and thimet oligopeptidase mainly cleaving after proline residues (see also Supporting Table S1). The branch containing the latter two proteases is not as clearly separated from other proteases when compared to the protease specificity tree based on the substrate recognition over the whole binding site. The residual tree contains unspecific proteases of all catalytic types sorted by increasing subpocket-wise cleavage entropy within P1 and hence unspecific substrate cleavage.

- Mapping of Ligand Data

Finally, we mapped targets of known protease inhibitors from the ChEMBL database to the protease specificity trees. We chose benzamidine (ChEMBL20936) as a well-studied protease inhibitor that occupies only a single protease subpocket S1 in bound state (e.g. [60]). We mapped known targets to the protease specificity tree based on S4-S1 amino acid frequencies (see Figure 6). Despite the wide usage of benzamidine as protease inhibitor in biochemistry (e.g. [61]), ChEMBL only lists bioactivity data for three protease targets in our test set. All three proteases plasmin, trypsin 1, and thrombin are serine proteases of the chymotrypsin fold known to prefer positively charged amino acids at P1 position and hence nicely group in one branch of the protease specificity tree.

Several ligands in ChEMBL are annotated to bind to even more than three different proteases. We chose BI 201335 (ChEMBL1241348) as example for a promiscuous non-covalent protease ligand inhibiting a wide range of proteases even distributed over different catalytic types. BI 201335 is a known inhibitor of the Hepatitis C Virus NS3-NS4A protease [62]. Mapping all 21 annotated targets in our protease set to the protease specificity tree over the whole binding site region, we observe a clustering of all known protease targets in particular branches of the degradome. Similarity between these targets is not observed on sequence or structure basis, as targets span all four major catalytic types of proteases.

Discussion

We present a novel approach to intuitively map the degradome based on substrate readout rather than protease sequence. The underlying methodology to construct a similarity matrix is solely based on subpocket amino acid frequencies, the same information visualized in common sequence logos of protease substrates. Therefore, the presented method is suitable for comparison of any kind of position-specific scoring matrix, e.g., a multiple sequence alignment or sequence motif. We encourage the community to use our method for comparison of sequence logos also in research apart from the protease universe.

Navigating the protease space using the approach described here has three major advantages over a protein sequence-driven view: First, protease similarity is inherently captured in the interaction with a substrate. Accordingly, binding site similarities are directly probed by using substrate data. Natural amino acids contain a variety of chemical features and provide multiple anchor points for interactions. By mapping known targets of small molecules to our protease specificity trees, we can directly translate knowledge from peptide and protein substrates to drug design of small molecules. Secondly, our approach is not limited to the analysis of similarities and differences between homologous proteases as sequence-based analyses. Taking the common feature, the cleaved substrates as basis, we are able to compare proteases of different

evolutionary origin. Therefore, we can engraft individual evolutionary trees of proteases to a complete map of the degradome. Finally, with increasing knowledge and availability of large scale data in protease databases, our data-driven mapping of the degradome can be more and more refined to result in a highly detailed view of protease substrate recognition. An annotation of true negatives, whether in the field of small molecule binding data or protease substrate data, would be especially helpful to refine current models. Likewise, quantitative data of binding affinity and kinetics could provide new insights into protease substrate recognition.

The presented principal component analysis on the similarity matrix of 62 proteases highlights the most important features of protease substrate recognition. The main variance in the data set results from substrate promiscuity quantified as total cleavage entropy and covered in PC1 and PC2. PC2 especially correlates with specificity of S1-P1 interactions, the major specific interaction point for most proteases, directly adjacent to the scissile bond. Further principal components read special subpocket interactions and hence group proteases by catalytic types, rendering a complex picture of protease-substrate recognition characteristics. This close interplay of protease catalytic types, evolutionary relations and diversification of specificity and function has been discussed over years (e.g. [63]), and are recovered by the statistical analysis presented in this study.

Analysis of protease-ligand annotations within ChEMBL shows a striking promiscuity of small molecules within the degradome. Broad binding profiles even overlap between catalytic types of proteases, adding an additional layer of complexity to the understanding or protease specificity. Current protease assay panels are usually limited to proteases with the same catalytic mechanism (e.g. [64]). Therefore, promiscuous binding to proteases of other catalytic type but similar substrate preferences would not be detected within these assays. We strongly encourage to setup broader protease assay panels to further trace ligand promiscuity within the protease field. Availability of suitable data sets would be of high interest for academic research.

Within the protease set covered in our study we observe different exchange probabilities for chemically closely related residues. Cleavage profiles show large overlaps in substrate recognition between proteases preferring positively charged residues, arginine or lysine, such as several members of the chymotrypsin fold. Still, we do not observe similar overlaps amongst substrate spectra of proteases recognizing different negatively charged residues. Caspases and granzyme B are highly specific for aspartate residues, whereas glutamyl peptidase I predominantly binds glutamate residues at the S1 pocket.

As our analysis of protease similarities based on cleaved substrates directly uncovers similarities in substrate recognition, we propose to apply our methodology for the prediction of off-target effects and understanding of polypharmacology within the protease field. Figure 6 shows how benzamidine binds to a branch of the protease specificity tree, whilst other members are omitted. We assume that benzamidine would also bind to the other proteases recognizing highly similar peptide substrates, e.g., granzyme A. This similarity in peptide binding is captured in our substrate-driven trees but not directly visible from sequence or structure due to different evolutionary origins. Still, more and more ligands binding to multiple similar but non-homologous binding sites are described in the literature [65]. Intuitively, drug repurposing efforts within the field of proteases can directly be based on our study via capturing substrate similarity.

Current strategies to predict or probe off-target effects include analysis of similarities in ligand structure, target structure as well as combinations thereof [66, 67, 68]. Especially, three-dimensional information has been described to be crucial in this

field [69]. Computational techniques applied to capture target structure similarity include molecular docking [70] as well as pharmacophore-based approaches [71]. Similar binding sites are expected to result in polypharmacology as a consequence of binding of similar ligands [72, 73]. Apart from prediction of polypharmacology, ligand-based network analyses have recently been found useful in the identification of unknown mechanisms of action of known drugs [74]. Our presented study introduces a novel ligand-based methodology to the field, the comparison of enzymes based on their peptide substrates.
Keeping in mind that substrate promiscuity is a general prerequisite for drug design [75], analyses of substrate promiscuity and specificity are of high importance for the protease field. Larger analyses of binding site similarities of protease mainly cover structure-based comparisons [48, 76], but neglect existing information on substrates. Following the general trend towards drug polypharmacology [77, 78] and the high potential of multi target drugs [79], we think that our study is an important step to fill that particular gap. By mapping the degradome from the perspective of substrates, similarities of protein binding sites can be captured directly. We have shown the straightforward applicability of information from peptide substrates in the chemical space of drug molecules and expect that similar studies are feasible for all kinds of protein-protein interfaces where sufficient substrate data is available.

References

1. Quesada V, Ordonez GR, Sanchez LM, Puente XS, Lopez-Otin C (2009) The Degradome database: mammalian proteases and diseases of proteolysis. Nucleic Acids Res 37: D239-D243.
2. Puente XS, Sanchez LM, Overall CM, Lopez-Otin C (2003) Human and Mouse Proteases: A Comparative Genomic Approach. Nat Rev Genet 4: 544-558.
3. Hedstrom L (2002) Introduction: Proteases. Chem Rev 102: 4429-4429.
4. Fuchs JE, Von Grafenstein S, Huber RG, Margreiter MA, Spitzer GM, Wallnoefer HG, Liedl KR (2013) Cleavage Entropy as Quantitative Measure of Protease Specificity. PLOS Comput Biol 9: e1003007.
5. Di Cera E (2009) Serine Proteases. IUBMB Life 61:510-515.
6. Lopez-Otin C, Overall CM (2002) Protease degradomics: a new challenge for proteomics. Nat Rev Mol Cell Biol 3: 509-519.
7. Turk B (2006) Targeting proteases: successes, failures and future prospects. Nat Rev Drug Discov 5: 785-799.
8. Schechter I, Berger A (1967) On the Size of the Active Site in Proteases: I. Papain. Biochem Biophys Res Commun 2: 157-162.
9. Madala PK, Tyndall JDA, Nall T, Fairlie DP (2010) Update 1 of: Proteases Universally Recognize Beta Strands In Their Active Sites. Chem Rev 110: PR1-PR31.
10. Perona JJ, Craik CS (1995) Structural basis of substrate specificity in the serine proteases. Protein Sci 4: 337-360.
11. Hedstrom L (2002) Serine Protease Mechanism and Specificity. Chem Rev 102: 4501-4523.
12. Rawlings ND, Barrett AJ, Bateman A (2012) MEROPS: the database of proteolytic enzymes, their substrates and inhibitors. Nucleic Acids Res 40: D343-350. Database accession 8.5.2013
13. Puente XS, Sanchez LM, Gutierrez-Fernandez A, Velasco G, Lopez-Otin C (2005) A genomic view of the complexity of mammalian proteolytic systems. Biochem Soc Trans 33: 331-334.
14. Lopez-Otin C, Bond JS (2008) Proteases: Multifunctional Enzymes in Life and Disease. J Biol Chem 283: 30433-

30437.

15. Poreba M, Drag M (2010) Current strategies for probing substrate specificity of proteases. Curr Med Chem 17: 3968–3995.
16. O'Donoghue AJ, Eroy-Reveles AA, Knudsen GM, Ingram J, Zhou M et al (2012) Global identification of peptidase specificity by multiplex substrate profiling. Nat Methods 9: 1095-1100.
17. Matthews DJ, Wells JA (1993) Substrate Phage – Selection of Protease Substrates by Monovalent Phage Display. Science 5111: 1113-1117.
18. Boulware KT, Daugherty PS (2006) Protease specificity determination by using cellular libraries of peptide substrates (CliPS). Proc Nat Acad Sci U S A 20: 7583-7588.
19. Turk BE, Huang LL, Piro ET, Cantlay LC (2001) Determination of protease cleavage site motifs using mixture-based oriented peptide libraries. Nat Biotechnol 19: 661-667.
20. Harris JL, Backes BJ, Leonetti F, Mahrus S, Ellman JA et al (2000) Rapid and general profiling of protease specificity by using combinatorial fluorogenic substrate libraries. Proc Natl Acad Sci U S A 14:7754-7759.
21. Mahrus S, Trinidad JC, Barkan DT, Sali A, Burlingame et al (2008) Global Sequencing of Proteolytic Cleavage Sites in Apoptosis by Specific Labeling of Protein N Termini. Cell 134: 866-876.
22. Rawlings ND, Barrett AJ, Bateman A (2010) MEROPS: the peptidase database. Nucleic Acids Res 38: D227-233.
23. Rawlings ND (2009) A large and accurate collection of peptidase cleavages in the MEROPS database. Database (Oxford) 2009: bap015.
24. Schneider TD, Stephens RM (1990) Sequence logos: a new way to display consensus sequences. Nucleic Acids Res 20: 6097-6100.
25. Schilling O, Overall CM (2008) Proteome-derived, database-searchable peptide libraries for identifying protease cleavage sites. Nat Biotechnol 6: 685-694.
26. Song J, Tan H, Perry AJ, Akutsu T, Webb GI et al (2012) PROSPER: An Integrated Feature-Based Tool for Predicting Protease Substrate Cleavage Sites. PLOS One 7: e50300.
27. Gaulton A, Bellis LJ, Bento AP, Chambers J, Davies M et al (2011) ChEMBL: a large-scale bioactivity database for drug discovery. Nucleic Acids Res 40: D1100-1107. Database accession 15.5.2013
28. Igarashi Y, Eroshkin A, Gramatikova S, Gramatikoff K, Zhang Y et al. (2007) CutDB: a proteolytic event database. Nucleic Acids Res 35: D546-D549.
29. Igarashi Y, Heureux E, Doctor KS, Talwar P, Gramatikova S et al. (2009) PMAP: databases for analyzing proteolytic events and pathways. Nucleic Acids Res 37: D611-D618.
30. Crooks GE, Hon G, Chandonia JM, Brenner SE (2004) WebLogo: A Sequence Logo Generator. Genome Res 14: 1188-1190.
31. McCaldon P, Argos P (1988) Oligopeptide Biases in Protein Sequences and Their Use in Predicting Protein Coding Regions in Nucleotide-Sequences. Proteins 2: 99-122.
32. Jones E, Oliphant T, Peterson P et al (2001) SciPy: Open Source Scientific Tools for Python. http://www.scipy.org
33. Rice P, Longden I, Bleasby A (2000) EMBOSS: The European Molecular Biology Open Software Suite. Trends Genet 6: 276-277.

34. Fitch WM, Margoliash E (1967) Construction of phylogenetic trees. Science 3760: 279-284.
35. Letunic I, Bork P (2011) Interactive Tree of Life v2: online annotation and display of phylogenetic trees made easy. Nucleic Acids Res 39 (Suppl 2): W475-W478.
36. Ekins S, Mestres J, Testa B (2007) In silico pharmacology for drug discovery: applications to targets and beyond. Br J Pharmacol 152: 21-37.
37. Rholam M, Fahy C (2009) Processing of peptide and hormone precursors at the dibasic cleavage sites. Cell Mol Life Sci 66: 2075-2091.
38. Lakhani SA, Masud A, Kuida K, Porter GA Jr, Booth CJ et al (2006) Caspases 3 and 7: Key Mediators of Mitochondrial Events of Apoptosis. Science 311: 847-851.
39. Thornberry NA, Rano TA, Peterson EP, Rasper DM, Timkey T et al (1997) A Combinatorial Approach Defines Specificities of Members of the Caspase Family and Granzyme B. J Biol Chem 272: 17907-17911.
40. Walsh JG, Cullen SP, Sheridan C, Luethi A, Gerner C et al (2008) Executioner caspase-3 and caspase-7 are functionally distinct proteases. Proc Natl Acad Sci U S A 105: 12815-12819.
41. Ng NM, Pierce JD, Webb GI, Ratnikov BI, Wijeyewickrema LC et al (2011) Discovery of Amino Acid Motifs for Thrombin Cleavage and Validation Using a Model Substrate. Biochemistry 50: 10499-10507.
42. Morrison CJ, Butler GS, Rodriguez D, Overall CM (2009) Matrix metalloproteinase proteomics: substrates, targets, and therapy. Curr Opin Cell Biol 21: 1-9.
43. Stegemann C, Didangelos A, Barallobre-Barreiro J, Langley SR, Mandal K et al (2013) Proteomic identification of matrix metalloproteinase substrates in the human vasculature. Circ Cardiovasc Genet 6: 106-117.
44. Checler F, Vincent JP, Kitabgi P (1986) Purification and Characterization of a Novel Neurotensin-degrading Peptidase from Rat Brain Synaptic Membranes. J Biol Chem 261: 11274-11281.
45. Cunha FM, Berti DA, Ferreira ZS, Klitzke CF, Markus RP et al (2008) Intracellular Peptides as Natural Regulators of Cell Signaling. J Biol Chem 283: 24448-24459.
46. Husson SJ, Clynen E, Baggerman G, Janssen T, Schofs L (2006) Defective processing of neuropeptide precursors in Caenorhabditis elegans lacking proprotein convertase 2 (KPC-2/EGL-3): mutant analysis by mass spectrometry. J Neurochem 98: 1999-2012.
47. Butler GS. Overall CM (2009) Updated Biological Roles for Matrix Metalloproteinases and New "Intracellular" Substrates Revealed by Degradomics. Biochemistry 48: 10830-10845.
48. Pirard B (2007) Insight into the structural determinants for selective inhibition of matrix metalloproteinases. Drug Discov Today 12: 640-646.
49. Deng SJ, Bickett DM, Mitchell JL, Lambert MH, Blackburn RK (2000) Substrate Specificity of Human Collagenase 3 Assessed Using a Phage-displayed Peptide Library. J Biol Chem 275: 31422-31427.
50. Syntichaki P, Xu K, Driscoli M, Tavernarakis N (2002) Specific aspartyl and calpain proteases are required for neurodegeneration in C. elegans. Nature 419: 939-944.
51. Williamson AL, Lecchi P, Turk BE, Choe Y, Hotez PJ et al (2004) A Multi-enzyme Cascade of Hemoglobin Proteolysis in the Intestine of Blood-feeding Hookworms. J Biol Chem 279: 35950-35957.
52. Krem MW, Di Cera E (2001) Molecular markers of serine protease evolution. EMBO J 20: 3036-3045.

53. Rose T, Di Cera E (2002) Substrate Recognition Drives the Evolution of Serine Proteases. J Biol Chem 277: 19243-19246.
54. Yousef GM, Kopolovic AD, Elliott MB, Diamandis EP (2003) Genomic overview of serine proteases. Biochem Biophys Res Commun 305: 28-36.
55. Debela M, Beaufort N, Magdolen V, Schechter NM, Craik CS et al (2008) Structures and specificity of the human kallikrein-related peptidases KLK 4, 5, 6, and 7. Biol Chem 389: 623-632.
56. Rotonda J, Nicholson DW, Fazil KM, Gallant M, Gareau Y et al (1996) The three-dimensional structure of apopain/CPP32, a key mediator of apoptosis. Nat Struct Biol 3: 619-625.
57. Rotonda J, Garcia-Calvo M, Bull HG, Geissler WM, McKeever BM et al (2001) The three-dimensional structure of human granzyme B compared to caspase-3, key mediators of cell death with cleavage specificity for aspartic acid in P1. Chem Biol 8: 357-368.
58. Wang B, Malik R, Nigg EA, Koerner R (2008) Evaluation of the Low-Specificity Protease Elastase for Large-Scale Phophoproteome Analysis. Anal Chem 80: 9526-9533.
59. Timmer JC, Zhu W, Pop C, Regan T, Snipas SJ et al (2009) Structural and kinetic determinants of protease substrates. Nat Struct Mol Biol 16: 1101-1108.
60. Buch I, Giorgino T, De Fabritiis G (2011) Complete reconstruction of an enzyme-inhibitor binding process by molecular dynamics simulations. Proc Natl Acad Sci U S A 108: 10184-10189.
61. Deutscher MP (1990) Maintaining protein stability. Methods Enzymol 182: 83-89.
62. White PW, Llinas-Brunet M, Amad M, Bethell RC, Bolger G et al (2010) Preclinical Characterization of BI 201335, a C-terminal Carboxylic Acid Inhibitor of the Hepatitis C Virus NS3-NS4A Protease. Antimicrob Agents Chemother 54: 4611-4618.
63. Neurath H (1984) Evolution of Proteolytic Enzymes. Science 224: 350-357.
64. Collet BYM, Nagashima T, Yu MS, Pohl NLB (2009) Fluorous-based Peptide Microarrays for Protease Screening. J Fluor Chem 130: 1042-1048.
65. Skolnick J, Gao M (2013) Interplay of physics and evolution in the likely origin of protein biochemical function. Proc Natl Acad Sci U S A 110: 9344-9349.
66. Keiser MJ, Roth BL, Armbruster BN, Ernsberger P, Irwin JJ et al (2007) Relating protein pharmacology by ligand chemistry. Nat Biotechnol 25: 197-206.
67. Hert J, Keiser MJ, Irwin JJ, Oprea TI, Shoichet BK (2008) Quantifying the Relationship among Drug Classes. J Chem Inf Model 48: 755-765.
68. Lounkine E, Keiser MJ, Whitebread S, Mikhailov D, Hamon J et al (2012) Large Scale Prediction and Testing of Drug Activity on Side-Effect Targets. Nature 486: 361-367.
69. Yera EA, Cleves AE, Jain AN (2011) Chemical Structural Novelty: On-Targets and Off-Targets. J Med Chem 54: 6771-6785.
70. Durrant JD, Amaro RE, Xie L, Urbaniak MD, Ferguson MAJ et al (2010) A Multidimensional Strategy to Detect Polypharmacological Targets in the Absence of Structural and Sequence Homology. PLOS Comput Biol 6: e1000648.

71. Liu X, Ouyang S, Yu B, Liu Y, Huang K et al (2010) PharmMapper server: a web server for potential drug target identification using pharmacophore mapping approach. Nucleic Acids Res 38: W609-W614.
72. Milletti F, Vulpetti A (2010) Predicting Polypharmacology by Binding Site Similarity: From Kinases to the Protein Universe. J Chem Inf Model 50: 1418-1431.
73. Vulpetti A, Kalliokoski T, Millerri F (2012) Chemogenomics in drug discovery: computational methods based on the comparison of binding sites. Future Med Chem 4: 1971-1979.
74. Gregori-Puigjane E, Setola V, Hert J, Crews BA, Irwin JJ et al (2012) Identifying mechanism-of-action targets for drugs and probes. Proc Natl Acad Sci U S A 109: 11178-11183.
75. Nobeli I, Favia AD, Thornton JM (2009) Protein promiscuity and its implications for biotechnology. Nat Biotechnol 27: 157-167.
76. Gold ND, Deville K, Jackson RM (2007) New opportunities for protease ligand-binding site comparisons using SitesBase. Biochem Soc Trans 35: 561-565.
77. Overington JP, Al-Lazikani B, Hopkins AL (2006) How many drug targets are there? Nat Rev Drug Discov 5: 993-996.
78. Yildirim MA, Goh KI, Cusick ME, Barabasi AL, Vidal M (2007) Drug-target network. Nat Biotechnol 25: 1119-1126.
79. Lu JJ, Pan W, Hu YJ, Wang YT (2012) Multi-Target Drugs: The Trend of Drug Research and Development. PLOS One 7: e40262.

Figure Legends

Figure 1:

Workflow followed in degradome mapping: Sequence logos of protease cleavage sites are extracted and combined to a vector containing probabilities of amino acids and each subpocket position. Thereby, quantitative distances between protease substrate readout can be calculated by scalar projection of one protease vector on the other.

Figure 2:

Principal component analysis of the protease similarity matrix: Eigenvectors of the protease similarity matrix are used to map the degradome in lower dimensionality. Plotting principal component 1 (PC1) versus principal component two (PC2) and coloring according to cleavage entropy in a spectrum from red (specific) via yellow to green (unspecific) (2a) shows that both primary principal components mainly contain information on protease specificity. Coloring according to catalytic types (2b, serine protease: cyan, metallo protease: pink, cysteine protease: dark grey, aspartic protease: blue, protease complex: white) shows that PC2 separates serine proteases from other degradome members. PC3 does not correlate to substrate promiscuity (2c), but rather splits up metallo proteases (2d). Similarly, PC6 does not correlate to overall substrate readout (2e), but groups catalytic types of proteases only via their substrate preferences in combination with PC3 (2f): Metallo proteases are grouped to the left, cysteine proteases on top, aspartic proteases on the bottom, serine proteases in the center.

Figure 3:

Protease specificity tree over whole binding site region: The degradome is mapped to a protease specificity tree based on substrate similarity over S4-S4'. Proteases are colored according to their catalytic type: serine proteases (cyan), metallo proteases (pink), cysteine proteases (dark grey), aspartic proteases (blue). The outer ring shows total cleavage entropies in a color spectrum from red (specific) over yellow to green (unspecific). The protease specificity tree shows striking similarities in substrate readout of proteases based on different catalytic mechanism.

Figure 4:

Protease specificity tree over the non-prime binding site region S4-S1: The degradome is mapped to a protease specificity tree based on local substrate similarity over S4-S1 pockets. Proteases are colored according to their catalytic type: serine proteases (cyan), metallo proteases (pink), cysteine proteases (dark grey), aspartic proteases (blue). The outer ring shows cleavage entropies for the range S4-S1 in a color spectrum from red (specific) over yellow to green (unspecific). The reduced scattering of catalytic types when compared to the protease specificity tree for the whole binding site indicates a grouping of evolutionary close members.

Figure 5:

Protease specificity tree based on S1 amino acids: The degradome is mapped to a protease specificity tree based on S1 amino acid frequencies in substrates. Proteases are colored according to their catalytic type: serine proteases (cyan), metallo proteases (pink), cysteine proteases (dark grey), aspartic proteases (blue). The outer ring shows subpocket cleavage

entropies for the S1 pocket in a color spectrum from red (specific) over yellow to green (unspecific). A grouping of proteases recognizing aspartic acid, basic amino acids as well as hydrophobic or unspecific proteases is observed.

Figure 6: Mapping of known targets of benzamidine to the substrate-driven protease specificity tree: Known targets from the ChEMBL database (outer ring blue) cluster on top of the protease specificity tree based on S4-S1 substrate readout compared to unknown targets (outer ring light grey) and targets without ChEMBL identifier (outer ring white). Proteases are colored according to their catalytic type: serine proteases (cyan), metallo proteases (pink), cysteine proteases (dark grey) and aspartic proteases (blue). Targets of benzamidine are members of the chymotrypsin fold preferring positively charged amino acids at P1. Off-target binding of benzamidine to proteases positioned in vicinity of the already known targets (e.g. granzyme A) is proposed.

Figure 7:

Mapping of known targets of BI 201335 to the protease specificity tree: Known targets in ChEMBL (outer ring blue) cluster on the right side of the protease specificity tree, calculated over the whole S4-S4' region, compared to unknown targets (outer ring light grey). Proteases without a ChEMBL identifier are colored white in the outer ring. Known targets include all catalytic mechanisms of proteases: serine proteases (cyan), metallo proteases (pink), cysteine proteases (dark grey) and aspartic proteases (blue). This highlights the promiscuous binding of a single ligand to several proteases.

Table S1:

List of 62 investigated proteases annotated with MEROPS and ChEMBL identifiers as well as catalytic types, number of substrates and sequence logo. Proteases are sorted according to MEROPS identifier to assure grouping of evolutionary branches.

Figure1
Click here to download high resolution image

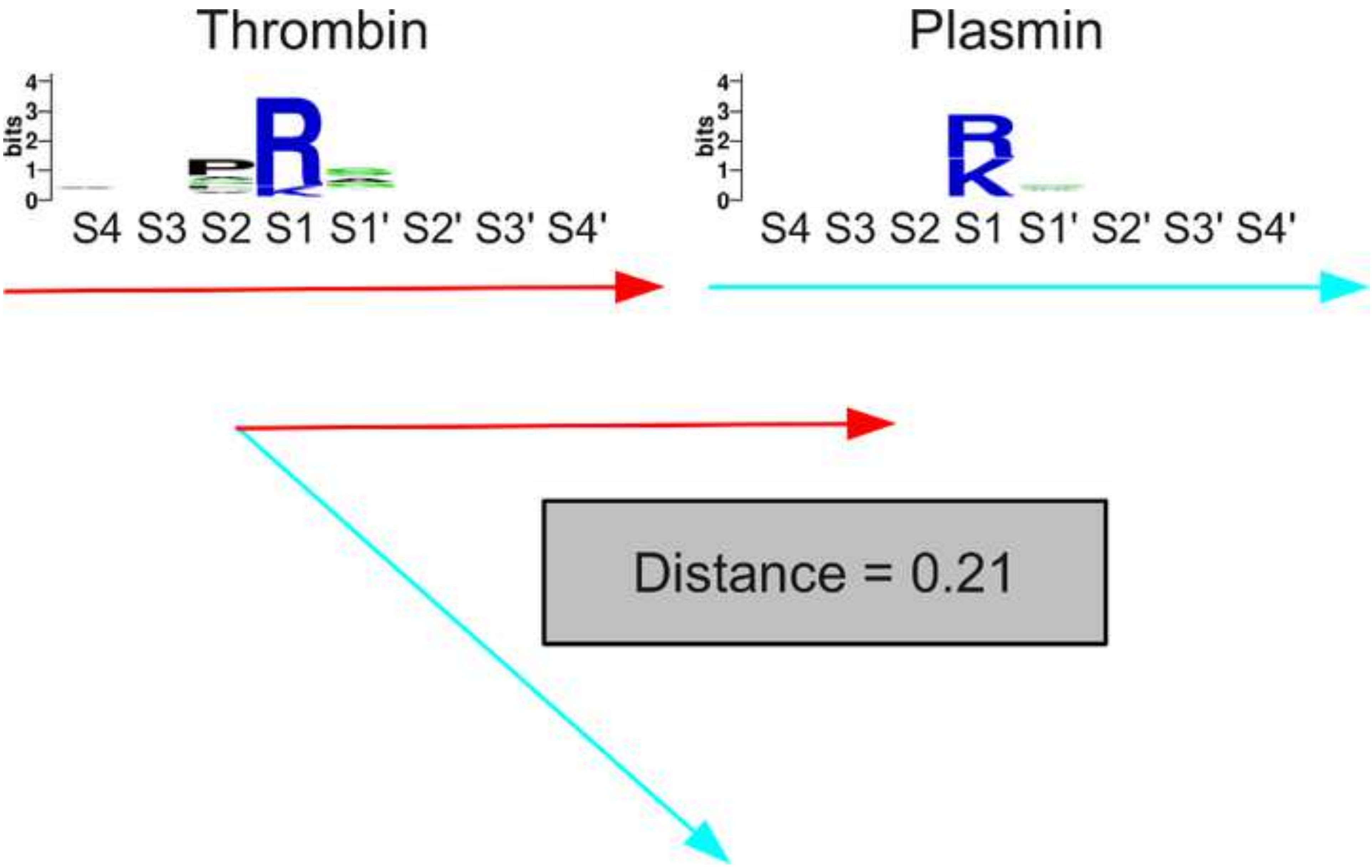

Figure2

Click here to download high resolution image

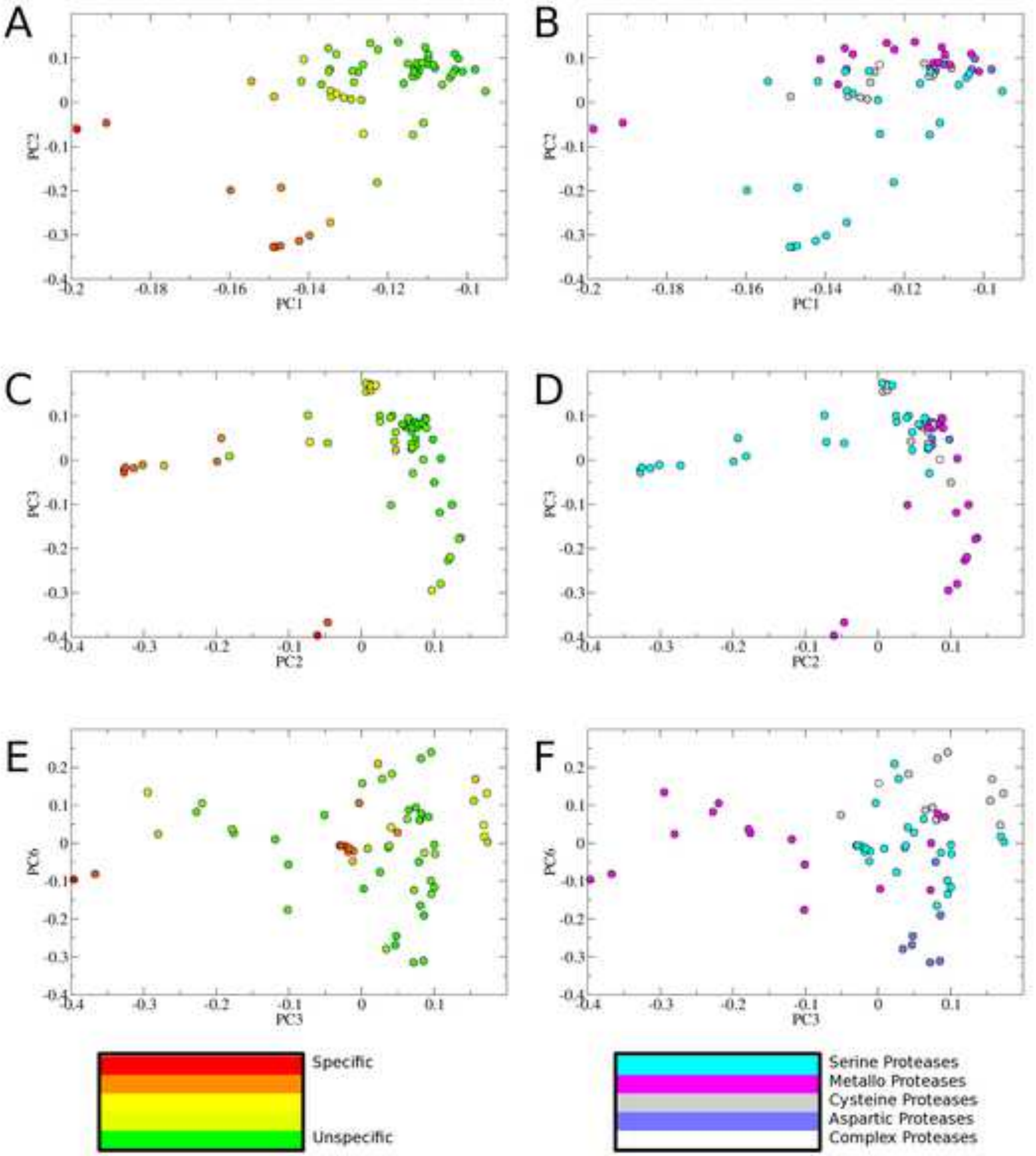

Figure3
Click here to download high resolution image

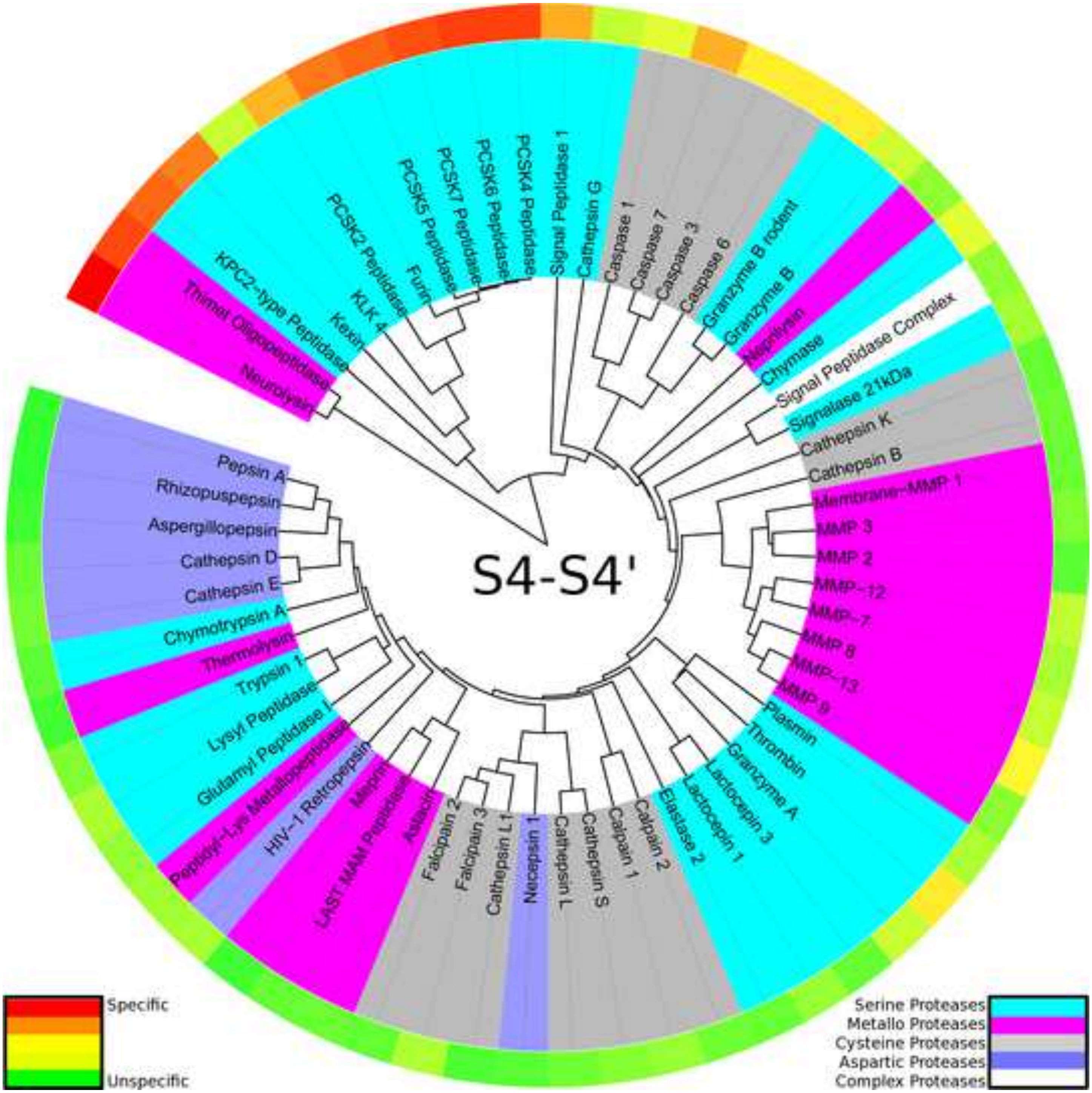

Figure4

Click here to download high resolution image

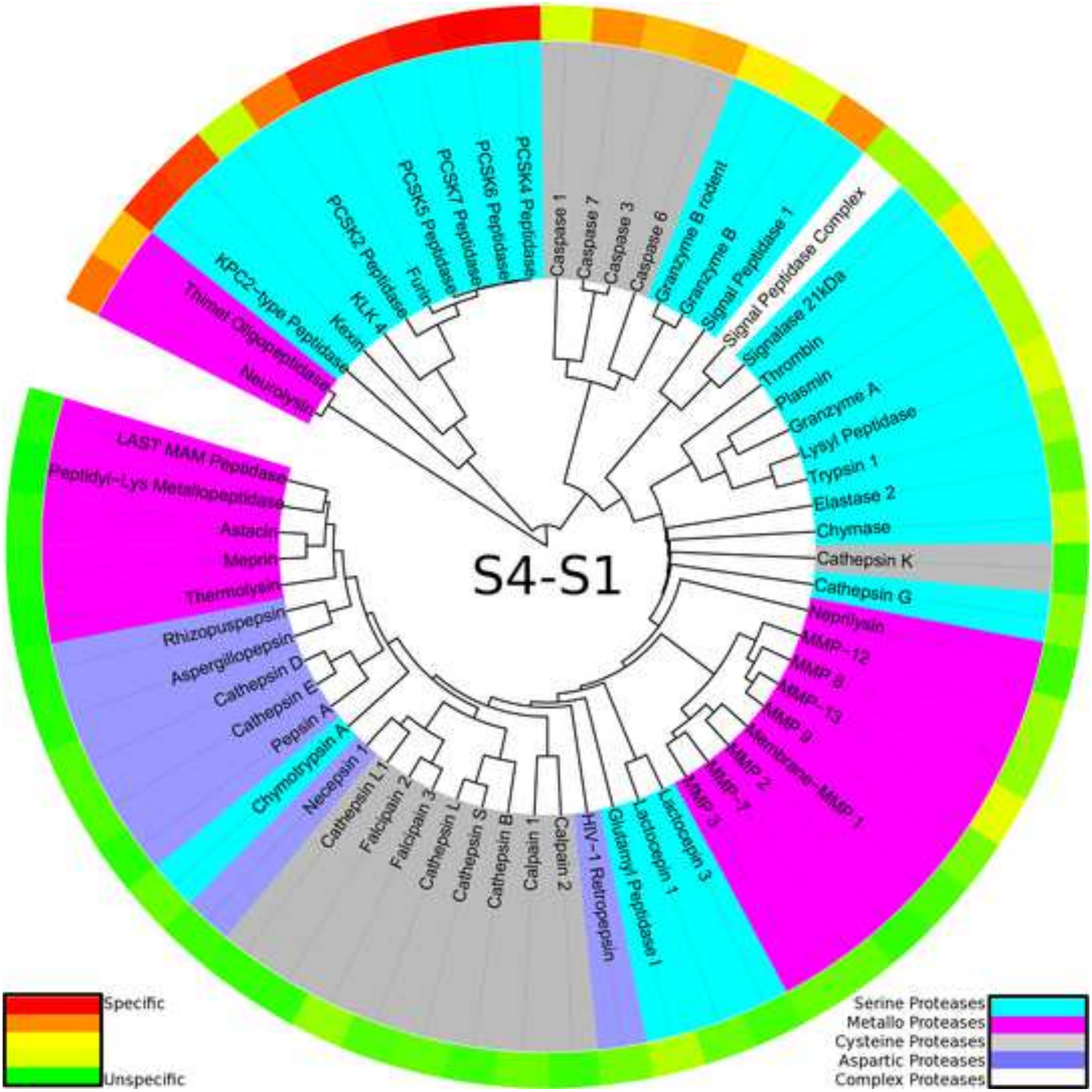

Figure5
Click here to download high resolution image

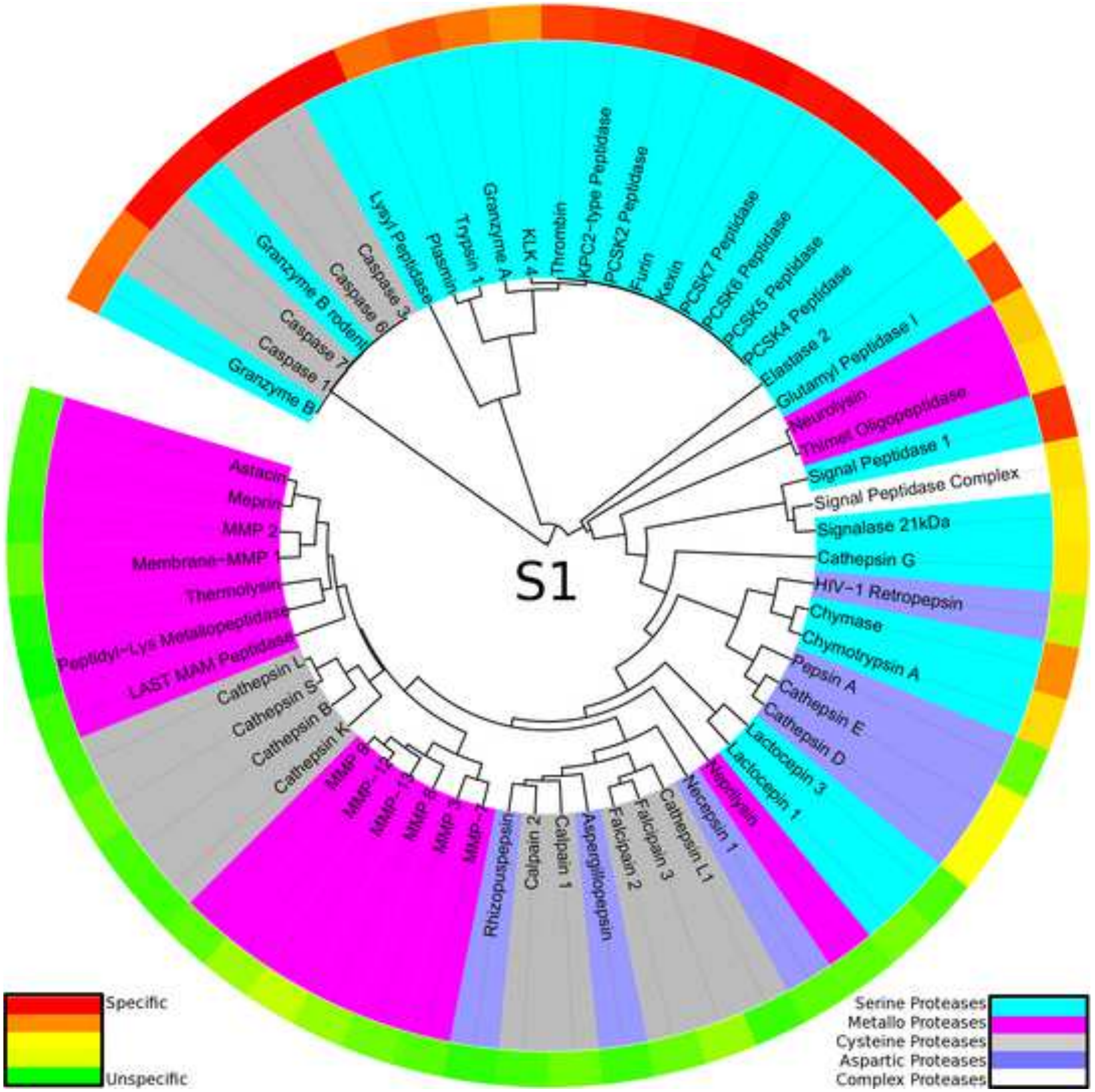

Figure6
Click here to download high resolution image

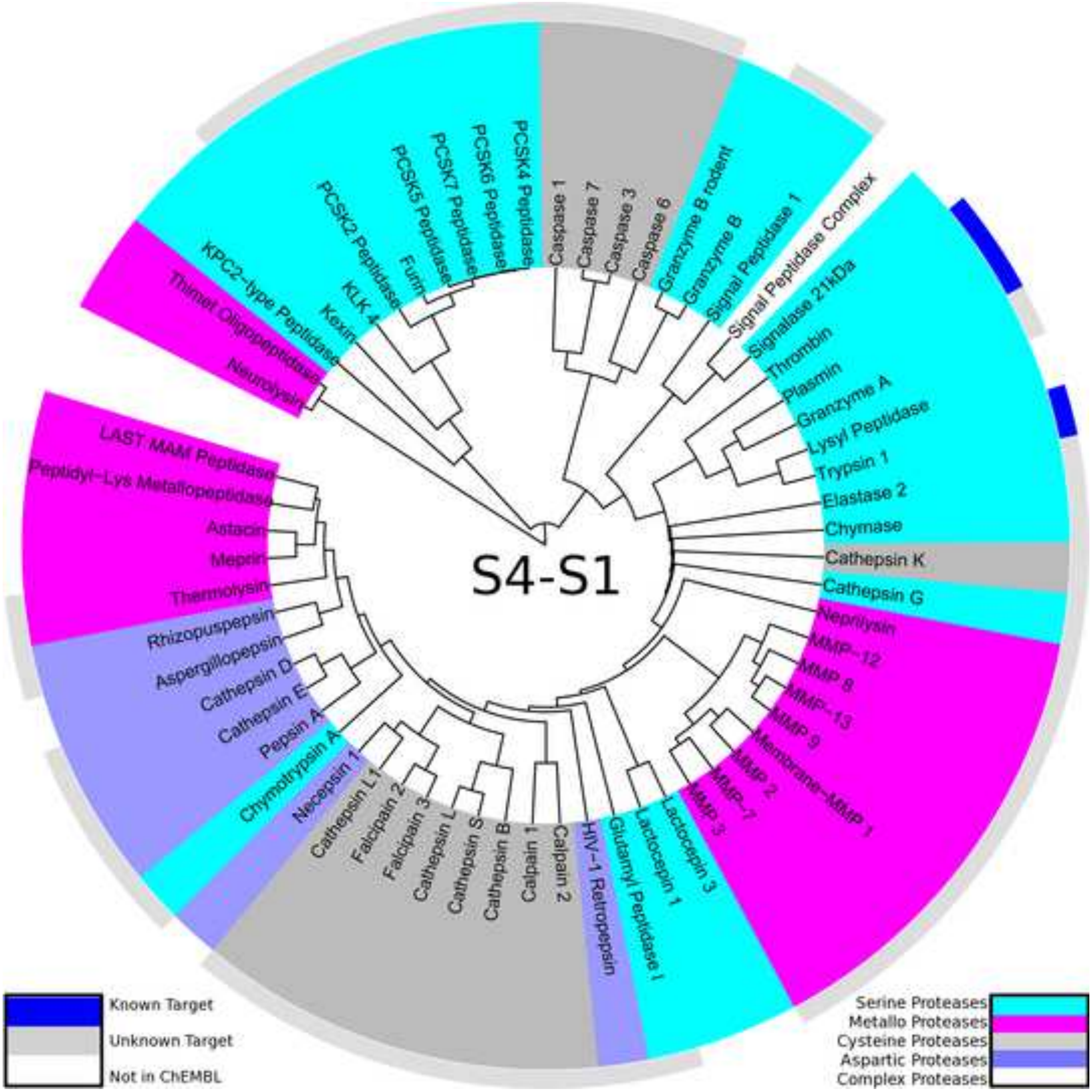

Figure7
Click here to download high resolution image

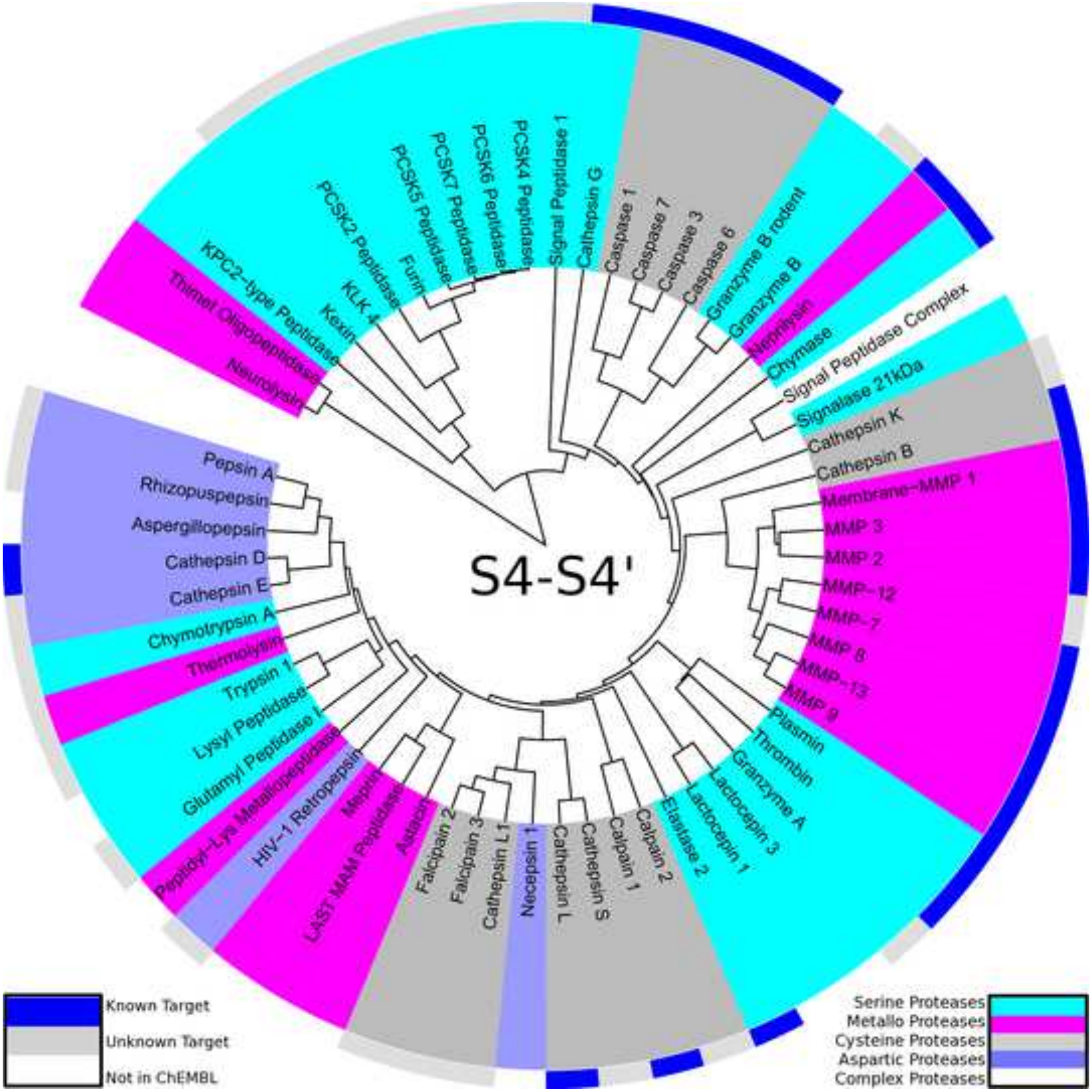

Supporting Information
Click here to download Supporting Information: TableS1.pdf
