# Supplementary material for: Specificity of a protein–protein interface: Local dynamics direct substrate recognition of effector caspases
Source: Proteins. 2014 Oct 19;82(4):546–55. doi: 10.1002/prot.24417 (PMC4282588; doi:10.1002/prot.24417)

SUPPORTING INFORMATION

Supporting Figure S1: 2D RMSD plots of 1000 frames periodically extracted over 50ns sampling time from simulations of caspase 3 holo and complex (top left) and caspase 7 holo and complex (top right). A comparison between both holo simulations and both complex simulations is shown in the lower line. A color gradient from violet over red to yellow indicates a maximum RMSD of 2.5 Å between snapshots sampled within all simulations. Hence, a comparable conformational space is sampled in four stable simulations, indicating a binding mechanism of conformational selection. 2D RMSD plots also show the described reduction of protein flexibility upon complexation with a substrate in the active site.

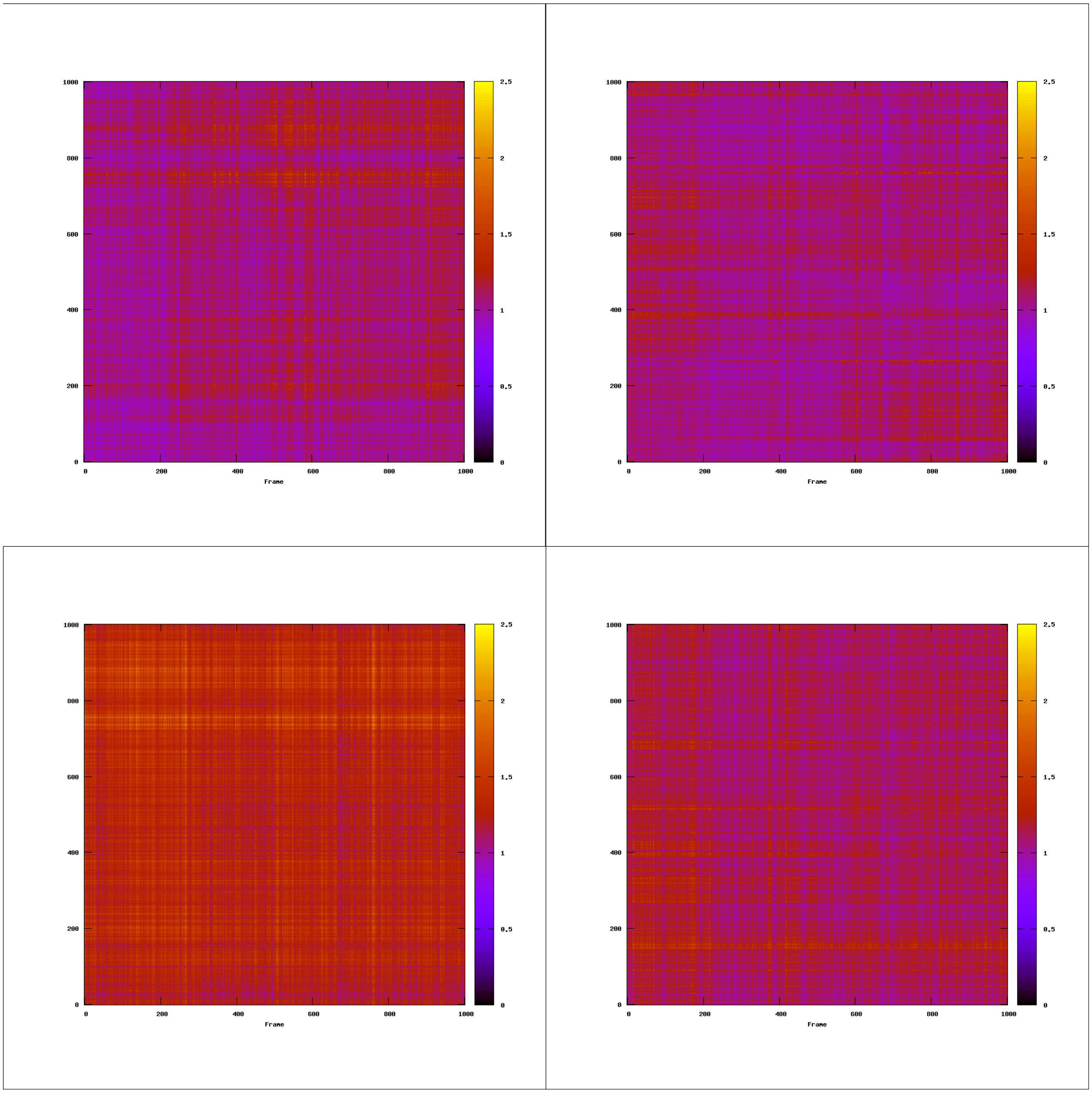

Supplement: Supplementary file 3 [file prot0082-0546-SD3.pdf]
